# WhatsApp-Supported Teledentistry to Reinforce Oral Health Promotion Among Older Adults Residing in Rural and Urban Areas: Randomized Controlled Trial

**DOI:** 10.2196/71251

**Published:** 2026-05-15

**Authors:** Fernanda Muñoz-Sepúlveda, Víctor Beltrán, Alfredo von Marttens, Pablo Navarro, Claudia Acevedo, Rodrigo Mariño, Alejandra Chaparro, Leonardo López

**Affiliations:** 1 Clinical Investigation and Dental Innovation Center (CIDIC), Dental School and Center for Translational Medicine (CEMT-BIOREN) Universidad de La Frontera Temuco Chile; 2 Interuniversity Center for Healthy Aging (CIES) Talca Chile; 3 Program of Master in Dental Science, Dental School Universidad de La Frontera Temuco, Araucanía Chile; 4 Department of Prosthodontics , Faculty of Dentistry University of Chile Santiago Chile; 5 Research Center for Dental Sciences (CICO), Dental School Universidad de La Frontera Temuco Chile; 6 Facultad de Ciencias de la Salud Universidad Autónoma de Chile Temuco Chile; 7 Melbourne Dental School University of Melbourne Melbourne Australia; 8 Center for Research in Epidemiology, Economics and Oral Public Health (CIEESPO), Faculty of Dentistry Universidad de La Frontera Temuco Chile; 9 Department of Periodontology, Centre for Biomedical Research, Faculty of Dentistry Universidad de Los Andes, Chile Santiago Chile; 10 Information and Communication Technology Institute Universidad de La Frontera Temuco Chile

**Keywords:** e-health, geriatric dentistry, health education, health inequities, health promotion, healthy aging, m-oral health, social media, teledentistry, telemedicine

## Abstract

**Background:**

Access to oral health promotion for older adults is globally limited, especially in rural, low- and middle-income settings. Digital research often lacks theoretical foundation and focuses primarily on younger cohorts, yielding few randomized trials evaluating accessible tools for oral health education in older adults.

**Objective:**

This study aimed to develop a telehealth reinforcement strategy for oral health promotion to improve knowledge, attitudes, and self-efficacy in community-dwelling older adults.

**Methods:**

A single-center, parallel-group randomized controlled trial was conducted in 4 municipalities (2 urban and 2 rural) in La Araucanía, Chile. Eligible participants were functionally independent adults aged ≥60 years with smartphone and internet access; those with cognitive impairment, complete edentulism, or inability to use WhatsApp were excluded. Participants were recruited from regional databases and assessed using the Geriatric Dental Specialties Tele-platform, a teledentistry tool for older adults. Participants were randomized (1:1) to face-to-face instruction (comparator) or the same instruction plus 2 weeks of social cognitive theory–informed telehealth reinforcement (4 validated videos via WhatsApp). Clinicians and statistical advisors were blinded. Primary outcomes (oral health knowledge, attitudes, and self-efficacy) were measured via telephone-administered questionnaires at baseline and 6 weeks post intervention. Secondary outcomes included acceptability and self-reported behaviors. Analyses included hypothesis testing, multiple correspondence analysis, and k-means clustering.

**Results:**

A total of 120 older adults were randomized (comparator: n=59; telehealth: n=61), with 103 analyzed (comparator: n=51; telehealth: n=52). Both groups showed substantial within-group improvements in oral health knowledge (comparator: Cohen *d*=0.93, 95% CI 0.52-1.34; *P*<.001; telehealth: Cohen *d*=1.07, 95% CI 0.66-1.48; *P*<.001) and self-efficacy (comparator: *r*=0.59, 95% CI 0.38-0.74; *P*<.001; telehealth: *r*=0.62, 95% CI 0.43-0.77; *P*<.001). In per-protocol analysis, telehealth improved dental caries knowledge (*P*=.03) and attitudes (*P*=.004), with no between-group differences in other domains (*P*>.05). In intention-to-treat analysis, telehealth showed a significant between-group difference for attitudes only (adjusted mean difference=0.91, 95% CI 0.34-1.48; *P*=.002), with no differences for overall oral health knowledge (*P*=.11) or self-efficacy (*P*=.59). Exploratory analyses indicated only the rural telehealth subgroup showed significant gains in attitudes (*P*=.003) and flossing (*P*<.001). Clustering suggested greater improvements among participants with higher baseline needs, predominantly rural, with fewer teeth. Telehealth demonstrated acceptability across multiple indicators (>80% for most measures) with no clinical adverse events; minor video-access issues occurred.

**Conclusions:**

Telehealth reinforcement provided significant advantages in oral health attitudes compared with face-to-face instruction. The intervention was acceptable and showed benefits among older adults with higher preventive needs, commonly seen in rural settings. By integrating theory-informed strategies into a familiar digital platform, this study adds evidence from rural and urban contexts, extending prior work on mobile oral health. It offers insights to address service gaps in underserved areas and highlights potential for feasible, context-aligned implementation. Future research should evaluate long-term effects, adaptability, and cost-effectiveness.

**Trial Registration:**

ClinicalTrials.gov NCT05917548; https://clinicaltrials.gov/study/NCT05917548

## Introduction

### Oral Health Context in Older Adults

As global life expectancy continues to rise, promoting healthy aging has become a critical public health priority, with the older population projected to more than double by 2050 [1]. However, significant oral health disparities persist among this population, who are disproportionately affected by preventable conditions—dental caries, periodontal disease, dry mouth, and tooth loss, among others [2-4]. 

Despite global efforts, significant disparities in oral health promotion remain, especially in low- and middle-income countries, where older adults face unique barriers such as limited access to health care and lower health literacy [5]. Additional barriers include insufficient health coverage, scarcity of tailored programs, declining overall health, and social determinants such as income, educational level, and rural residence [6-10]. Rural communities, home to 43% of the world’s population, are especially underserved [11,12]. In Chile, 14.1% of older adults live in rural areas—a proportion that continues to grow—highlighting the urgent need to address territorial inequities in oral health and to develop more equitable strategies [12,13].

### Oral Health Promotion and Strategies Based on Behavioral Change Theories

Health promotion empowers individuals to improve their well-being; health education is its core instrument for enhancing knowledge, motivation, and self-management skills [14]. Reinforced educational interventions can prevent disease, support well-being, and foster informed decision-making [15]. In oral health, educational interventions have shown to improve plaque control, gingival health, and salivary function by targeting knowledge, attitudes, and self-efficacy—key predictors of behavioral change [16-18].

By addressing psychosocial determinants, interventions based on psychological theories, such as Bandura’s social cognitive theory (SCT), can support behavioral change [19-21]. SCT emphasizes the role of self-efficacy, observational learning, and reinforcement in promoting behavior change [22]. Among older adults, fostering self-efficacy, the belief in one’s ability to perform a specific behavior to achieve a desired outcome, is key to promoting autonomy and long-term well-being, as it strengthens their physical abilities and fosters a sense of active participation in addressing daily challenges [23-25]. This psychological resource plays a pivotal role in influencing long-term oral health behaviors, linking knowledge and healthy actions such as toothbrushing and overall oral care, which are associated with healthy aging lifestyles [26-31].

### Innovations in Oral Health for Older Adults: The Role of Digital Technologies

Persistent inequities in health demand a fundamental shift in how care is conceptualized and delivered. Information and communications technologies (ICTs) play a central role in advancing several Sustainable Development Goals (3, 4, 5, 9, and 17) by strengthening health systems and promoting universal health coverage [32-34]. For older adults, ICTs offer notable benefits—such as improved access to care, fewer in-person visits, lower costs, and enhanced overall quality of life [35-39]. However, adoption remains uneven due to age-related barriers, including physical limitations, resistance to change, low digital literacy, and privacy concerns—challenges known collectively as the “gray digital divide” [37,40,41].

In dentistry, ICT-based solutions have improved knowledge, promoted self-care, facilitated early disease detection, and enabled timely referrals to specialized care [41]. Some oral health promotion strategies, known as telehealth education [42], have been implemented among community-dwelling older adults through web platforms, text messages, and mobile apps [31,43-45]. A promising innovation within this field is mobile oral health (m-oral health), which uses mobile devices to promote and maintain oral health [42]. This strategy could prove particularly effective as technology use among older adults is increasing, using it not only for communication but also to seek health-related information—such as diseases, symptoms, treatments, nutrition, and health promotion [46-48]. Current internet usage among this population ranges from 32% to 99%, with higher rates observed among individuals with greater educational attainment and those living in urban settings (91% vs 76% in rural areas) [13,49]. Since the COVID-19 pandemic, smartphone adoption has also grown, reaching 60% among adults aged 60 years and older, although adoption remains significantly lower (20%) among individuals aged 80 years and older [50,51]. Additionally, 55.5% of individuals aged 60 years and older report using social media platforms—such as WhatsApp, Facebook, YouTube, and Instagram—for both information and interaction [51]. Although still underexplored, mobile health (mHealth) and social media are gaining traction and hold great potential to support older adults, particularly as many seek additional health information after clinical encounters but often lack guidance on where to find it [52,53].

When integrated with telehealth education, social media allow on-demand access to instructional content, enhancing their comfort and independence [52,54]. WhatsApp in particular has been increasingly used in health education interventions, demonstrating positive outcomes and highlighting its potential for supporting chronic disease management and health promotion [55]. It has shown effectiveness in reinforcing behaviors related to medication adherence [56], chronic disease management [57], and increasing the uptake of cervical cancer screening [58]. In oral health, social media-based interventions have improved knowledge and reduced gingival index scores, mainly in younger cohorts [46]; extending these approaches to older adults offers a promising way to integrate tailored oral health promotion into daily routines, potentially enhancing their well-being and benefiting their families and communities [36].

Therefore, this randomized controlled trial aims to develop and evaluate a theory-informed telehealth educational reinforcement intervention delivered via WhatsApp, assessing its impact on oral health knowledge, attitudes, and self-efficacy among community-dwelling older adults living in rural and urban areas of the La Araucanía Region, Chile. We hypothesize that participants receiving the intervention will demonstrate greater improvements in these outcomes compared with the comparator group.

## Methods

### Ethical Considerations

Ethics approval was obtained from the Research Ethics Committee of the Universidad de La Frontera, Temuco, Chile (Folio 082/22), prior to study initiation. The study was conducted in accordance with institutional guidelines, national regulations, and the Declaration of Helsinki. Study activities began on March 3, 2023; participant enrollment occurred between March and August 2023, and the trial concluded on October 14, 2023. The trial was retrospectively registered at ClinicalTrials.gov (NCT05917548) on June 6, 2023, due to an administrative delay in completing the registry process. Primary and secondary outcomes and the statistical analysis plan were prespecified prior to participant enrollment and were not modified thereafter. No deviations from the original study protocol were made during the conduct of the trial. Although these documents were not publicly posted at that time, all prespecified methodological and analytical procedures are transparently and comprehensively reported in this manuscript.

Participants first provided verbal consent by phone to be contacted and screened for eligibility. All participants then signed written informed consent after explaining study procedures (educational activities, survey administration at 3 points), voluntary participation, the right to withdraw at any time, and potential benefits and harms of participation. The anticipated benefits included improvements in oral health knowledge, attitudes, and self-efficacy; however, to minimize bias, the specific study aims were withheld in accordance with the Council for International Organizations of Medical Sciences guideline 10 [59]. No physical harm was anticipated due to the noninvasive nature of the intervention; potential risks were limited to minor discomfort from survey completion or digital content delivery. Data were anonymized prior to analysis, and no financial or material incentives were provided. This study did not collect or include any identifiable images of participants; therefore, no image-related consent was required.

### Patient and Public Involvement

Participants were not involved in study design, conduct, or reporting; they participated only in the recruitment, consent, and data collection phases. The intervention procedures and outcomes were developed by the research team based on evidence and expert consensus in geriatric oral health and telehealth.

### Trial Design

This single-center, parallel-group, randomized controlled clinical trial used a 1:1 allocation ratio to compare the impact of 2 health education interventions: one delivered face-to-face and the other through ICT as an educational reinforcement. The trial followed a superiority framework to evaluate whether the ICT-based reinforcement produced greater improvements in oral health knowledge, attitudes, and self-efficacy than standard face-to-face education. The report follows the CONSORT (Consolidated Standards of Reporting Trials) 2025 statement and CONSORT-EHEALTH (Consolidated Standards of Reporting Trials of Electronic and Mobile Health Applications and Online Telehealth) checklist (Multimedia Appendix 1) [60,61].

### Trial Setting

The study was conducted in the La Araucanía Region in southern Chile, selected for its high proportion of population aged ≥60 years (18.9%) [62], and its combination of rurality and social vulnerability, which contribute to health inequities and low frequency of routine and preventive oral health care [9,63]. Through a presidential social grant, our team provided oral health care to 480 older adults in the Region via a teledentistry workflow [64,65]. Participants were recruited by telephone from the regional National Senior Citizen Service (Chile) branch and local community organization databases and invited if eligible to attend appointments at a mobile dental clinic in the municipalities of Temuco (the regional capital, urban), Angol (urban), Freire (rural), and Los Sauces (rural).

### Eligibility Criteria

The inclusion criteria were adults aged 60 years or older, of any gender, functionally independent (Barthel index ≥90), cognitively able to follow instructions, and with access to a smartphone with internet connectivity. Exclusion criteria included cognitive impairment (Mini-Mental State Examination score of <25), complete edentulism, or inability to use WhatsApp.

### Sample Size

Sample size was calculated using G*Power (version 3.1.9.7; Heinrich Heine University Düsseldorf) for Windows, to assess differences between 2 independent means with unequal variances. The calculation was based on a prior study comparing oral health knowledge gains in adolescents who received face-to-face health promotion (SD 0.66) versus those who received additional video reinforcement via mobile app (SD 0.52), showing a knowledge difference of 0.42 points and an effect size of 0.7 [66]. Given the differences between adolescents and older adults in terms of cognitive processing, health literacy, and digital engagement, a more conservative effect size of 0.5 was selected. With a significance level of 0.05 and a power of 0.80, the minimum required sample size was 102 participants. Accounting for potential attrition, and in line with average withdrawal rates in clinical trials [67], the final sample included 120 participants randomly assigned to each group.

Stratified random sampling was conducted based on age categories (60-69 years, 70-79 years, and ≥80 years) and geographic sector (urban/rural by province), adjusted based on the percentage of older adults with access to a smartphone within each age group [51].

### Randomization and Blinding

Participants were randomly allocated to the comparator or the telehealth education group in a 1:1 ratio using the EPIDAT random number generator (version 4.2) [68], stratified by age group. Randomization was managed by an independent researcher not involved in recruitment or intervention delivery. After confirming eligibility and obtaining written consent, the principal investigator contacted the independent researcher to receive each participant’s group allocation.

To prevent contamination and crossover, the general dentist, outcome assessor, and data analysts were blinded to group assignments. Although participants were aware of the intervention during the reinforcement phase, they signed an agreement committing not to share the videos with family or friends if they received them.

### Development of Educational Materials

A pilot study in rural La Araucanía (n=76) identified knowledge gaps that informed the development of 4 educational modules: self-care, dental caries, periodontal disease, and oral cancer. Guided by SCT, materials were designed to model desired behaviors, build behavioral capability, offer verbal persuasion from professionals, and present positive outcomes to foster self-efficacy [22]. Content followed clinical guidelines [69-72] and focused on oral hygiene practices (toothbrushing and flossing), disease prevention, and symptom recognition.

Quality was evaluated by 8 academic reviewers using the Item Content Validity Index (I-CVI≥0.83) and the Patient Education Materials Assessment Tool for Audiovisual Materials (adequacy threshold ≥70% for both understandability and actionability) [73,74]. Existing videos on oral hygiene and oral cancer prevention already met these criteria (understandability: 91% and 87%; actionability: 100% for both). Revisions improved readability, clarity of clinical terms, and the use of informative headings. Newly developed scripts on dental caries and periodontal disease achieved full content validation (I-CVI=1.0), except for the “periodontal disease risk factors” item (I-CVI=0.88). Final videos scored 97% and 98% in understandability and 100% in actionability for dental caries and periodontal disease, respectively [74].

Videos combined peer narratives and demonstrations by older adults with expert guidance from a dental specialist. A standardized Microsoft PowerPoint presentation was also developed to align with video content.

### Intervention and Comparator

#### Comparator Group (Face-to-Face Only)

Participants received a 15-minute individual session delivered by a trained general dentist. The session incorporated key SCT components including behavioral capability (clear instruction and live demonstrations of toothbrushing, flossing, and recognition of early oral disease signs), observational learning (dentist modeling and hands-on practice using a dental model and participants’ own mouths), verbal persuasion (personalized encouragement), self-efficacy (personalized feedback), and outcome expectations (discussion of benefits of preventive behaviors and potential consequences of untreated oral conditions). Environmental facilitation was provided through a standardized Microsoft PowerPoint presentation, protected instructional time, and direct professional support.

#### Telehealth Education Group

In addition to the face-to-face session, participants received 4 educational videos sent via WhatsApp over 2 weeks (2 videos per week, approximately 5 minutes each, linked to YouTube). Participants were asked to watch the videos and informed that their opinions would be requested later; however, no specific viewing instructions were provided. Videos reinforced SCT components by offering environmental facilitation (on-demand access to educational content), behavioral capability (clear demonstrations of practical oral hygiene skills, explanations of early signs and symptoms of oral diseases, and guidance on preventive behaviors and risk factor management), observational learning (peer modeling—older adults performing target behaviors such as toothbrushing, flossing, seeking dental care, inspecting the oral mucosa for signs of disease, choosing healthy foods, maintaining hydration, and avoiding risk factors like smoking). The intervention also emphasized mastery experience (indirectly by encouraging participants to try the behaviors themselves after viewing the demonstrations), verbal persuasion (motivational messages and actionable recommendations from dental professionals), and outcome expectations (positive results of maintaining oral health and the benefits associated with the recommended behaviors).

### Procedures

Participants were enrolled and managed using the Geriatric Dental Specialties Tele-platform (TEGO), which supported data collection and clinical evaluations. The study followed four phases: (1) enrollment and baseline (pretest) assessment conducted via telephone, (2) clinical evaluation and face-to-face education at a mobile dental clinic by a trained general dentist, (3) telehealth educational intervention via WhatsApp for the intervention group, and (4) follow-up assessments conducted by telephone 6 weeks post intervention.

### TEGO System

#### Overview

The TEGO is a web-based system supporting oral health assessment and education for older adults, integrating an electronic dental record and interactive 3D models. Its protocol and workflow are detailed in prior publications [64,75,76].

#### Module 1: Enrollment

Collects sociodemographic information, including age, sex, place of residence [77], socioeconomic status via the Social Registry of Households [78], educational level, and smartphone usage. It also serves as the entry point for administering questionnaires, detailed in the “Outcomes” section.

#### Module 2: Medical-Geriatric Anamnesis

Conducted by a dentist, assessing cognitive status (Mini-Mental State Examination), psychological well-being (Short Geriatric Depression Scale), functional capacity (Barthel Index and Fried’s Frailty Phenotype), and medical conditions (eg, multimorbidity, defined as the presence of 2 or more comorbidities [79]). All instruments are validated in Spanish and commonly used in primary care [80-83].

#### Module 3: Odontogeriatric Anamnesis

Collects self-reported oral health behaviors (eg, toothbrushing, flossing, sugar consumption, and dental service use) and performs clinical evaluation using a standardized 3D dental model to record the number and condition of teeth [76].

### Outcomes

#### Overview

Assessments were conducted at baseline and 6 weeks post intervention. Some secondary outcomes (eg, acceptability) were measured only at follow-up, while oral hygiene behaviors were first collected during the clinical evaluation and at follow-up. The independent variable was the type of educational intervention.

#### Primary Outcomes

The primary outcomes were changes in oral health knowledge, attitudes, and self-efficacy, measured using the 53-item Oral health Knowledge, Attitudes, and Self-Efficacy Questionnaire, previously used in older adults’ populations [17,30,31,84]. The instrument includes 3 components:

1. Knowledge (39 items): assessed understanding of dental caries, periodontal disease, and oral cancer. Items included dichotomous (yes/no) questions on signs and symptoms, and a 5-point Likert scale rating the importance of risk and preventive factors (“Very important” to “Not important at all”). Correct answers scored 1 point (maximum=39 points; subscores: caries 12, periodontal disease 14, oral cancer 13).
2. Attitudes toward oral health (7 items): evaluated beliefs about the inevitability of oral diseases in older age, perceptions of prevention, and general oral health beliefs. Responses were recorded on a 4-point Likert scale ranging from “Strongly agree” to “Strongly disagree,” with higher scores indicating more positive attitudes.
3. Oral Health Self-Efficacy Scale (OHSES): The original English version of the OHSES includes 8 items divided into 2 domains: oral health self-efficacy (6 items) and general health self-efficacy (2 items) [85]. The Spanish version of OHSES, validated in older Chilean adults [86], uses a 5-point Likert scale (“Not at all confident” to “Strongly confident”). It assesses the perceived ability to prevent tooth loss (3 items) and general self-care (4 items). The scale has shown high internal consistency (Cronbach α=0.82) and acceptable test-retest reliability (intraclass correlation coefficient=0.41) [86].

Change scores were compared between intervention groups and further stratified by place of residency (urban vs rural, based on national classification [77]).

#### Secondary Outcomes

1. Self-reported oral hygiene behaviors: frequency of toothbrushing and dental flossing.
2. Smartphone use: categorized into four increasing levels of complexity: (1) basic communication (calls and WhatsApp), (2) informative search (adds internet browsing), (3) extended social interaction (adds other social media), and (4) advanced use (adds email).
3. Acceptability of telehealth education (intervention group only): defined as the perceived appropriateness of the intervention [87], and assessed through: (1) burden: evaluated as the perceived effort required to participate, approximated through behavioral proxies: the proportion of eligible individuals who agreed to enroll (willingness to participate) and the dropout rate (number of withdrawals divided by the total randomized × 100). (2) Affective attitude: assessed participants’ feelings about the intervention, measured via a dichotomous posttest question on willingness to recommend the video-based program. (3) Perceived effectiveness: this construct examines the extent to which the intervention is perceived to achieve its purpose and was measured through the perceived use of each video using a 5-point Likert scale (“Extremely useful” to “Not useful at all”).

#### Harms

Given the noninvasive nature of the intervention, harms were defined as any adverse events or burdens related to participation in study procedures, including difficulties with digital content delivery, frustration associated with technology use, or discomfort during questionnaire completion. No physical harm was expected.

Harms were assessed nonsystematically, through participants’ reports during follow-up contact and at each assessment point. Study staff also monitored indications of discomfort or technical burdens during phone interactions.

### Statistical Methods

The per-protocol analysis was established as the primary approach, including only participants who completed all study procedures. Noncompleters were excluded from this analysis.

Descriptive statistics were computed for sociodemographic variables, clinical data, self-reported behaviors, and questionnaire variables and are presented as percentages, means, SDs, and 95% CIs. The oral health knowledge, attitudes, and self-efficacy questionnaire was also examined by domain. Normality was assessed using the Anderson-Darling and D’Agostino tests. Within-group changes were assessed using 2-tailed paired *t* tests or Wilcoxon signed-rank tests. Between-group posttest comparisons were analyzed via independent *t* tests or Mann-Whitney *U* tests, and further examined using analysis of covariance (ANCOVA), adjusting for baseline scores. Effect sizes (Cohen *d* or *r*), 95% CIs, and *P* values are reported.

Three supplementary post hoc analyses were conducted:

1. Sensitivity analysis: an intention-to-treat (ITT) analysis was performed to preserve the integrity of randomization and minimize attrition bias. Missing data were handled using multiple imputation based on fully conditional specification, with linear regression models applied to scale variables. Five imputed datasets were generated with 10 iterations each, assuming data were missing at random. Both within-group and between-group ANCOVA analyses were conducted. A complementary Worst-Case Reasonable Scenario analysis assessed robustness under potential missing not at random (MNAR) mechanisms, assigning noncompleters in the telehealth group their baseline score (assuming no benefit) and comparator noncompleters the 75th percentile score of completers (assuming maximal benefit). This dual approach evaluates the stability of primary findings across different missing data assumptions.
2. Exploratory subgroup analysis: post hoc subgroup analysis was conducted by place of residence (urban/rural), introduced to enhance the interpretability of differential responses across settings. This includes the baseline percentage of correct responses by disease category and within-group analysis.
3. A post hoc multiple correspondence analysis and k-means clustering were performed within the telehealth group to explore associations between pre-post score changes and sociodemographic (age, educational level, place of residence, and smartphone use) and clinical variables (functional dentition, defined as ≥20 teeth vs <20 teeth). Score differences were categorized into 3 levels based on thresholds reported in previous studies [30,31,84]: significant improvement (≥50% increase), moderate improvement (20%-49% increase), and no change or deterioration (<20% improvement or decrease).

Acceptability of the telehealth intervention was assessed descriptively using percentages for perceived usefulness, willingness to recommend, and dropout rates.

Harms were assessed descriptively, including any difficulties related to digital engagement with digital content or survey procedures.

Analyses were performed using IBM SPSS Statistics (version 23.0) with supplementary visualizations in GraphPad Prism (version 8; GraphPad Software). A *P* value of <.05 was considered statistically significant.

## Results

### Participant Flow and Recruitment

**Figure 1:**
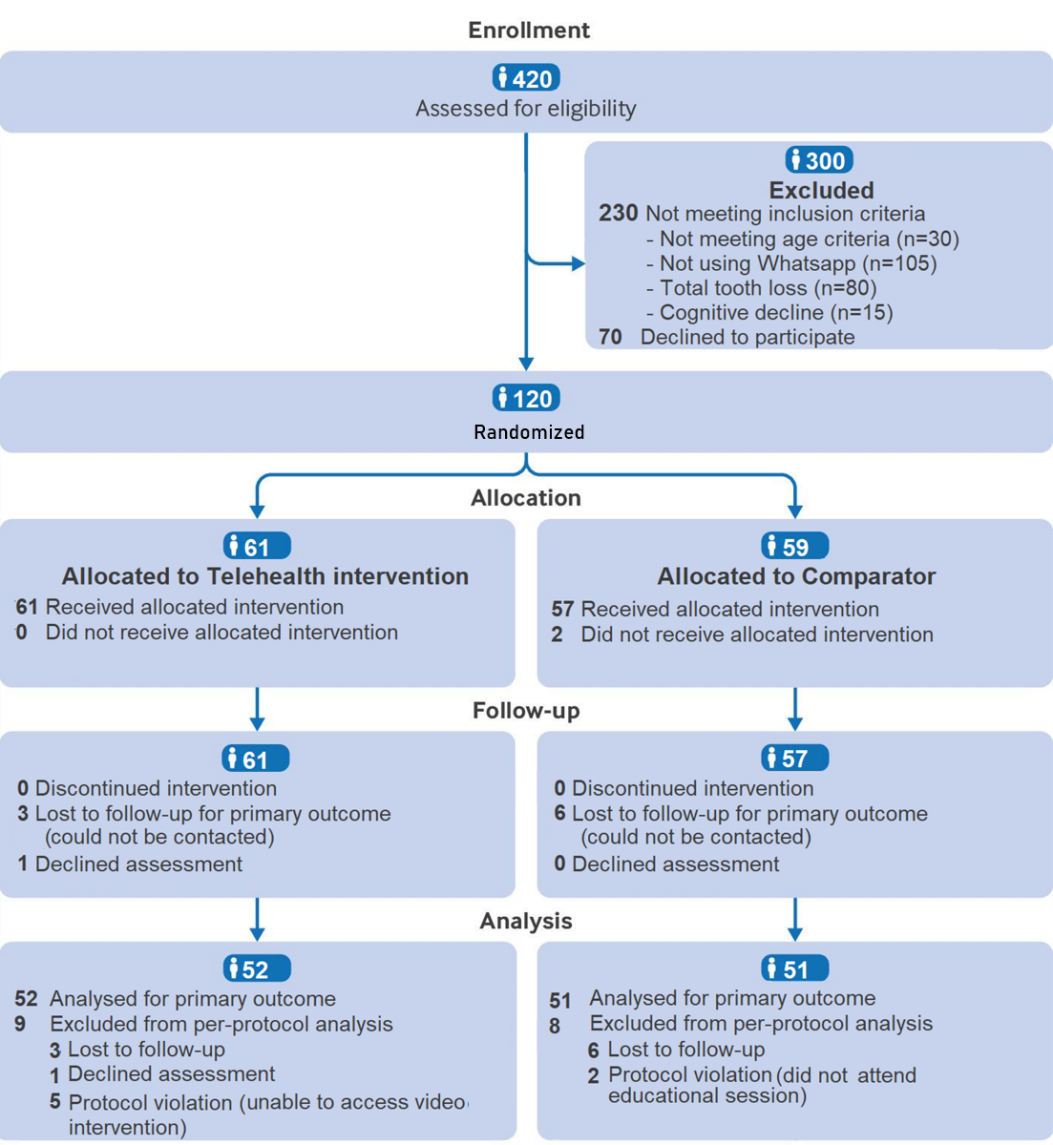
CONSORT (Consolidated Standards of Reporting Trials) 2025 flow diagram of the study showing participant enrollment, group allocation, follow-up, and analysis.

Between March 3 and August 4, 2023, a total of 120 participants were randomly assigned to the telehealth education group (n=61) or the comparator group (n=59; Figure 1). The last follow-up was completed on September 15, 2023. All participants provided informed consent prior to enrollment. Most participants received the intervention as intended; losses and reasons for exclusion after randomization are detailed in Figure 1. Both interventions were delivered as planned, with standardized procedures to ensure fidelity. Analyses followed a per-protocol approach and included only those who completed the study procedures (n=103), with 52 in the telehealth education group and 51 in the comparator group. No additional oral-health educational activities were provided during the trial period.

### Baseline Data

The median age of participants was 68  (IQR 63-73) years, and 64% (77/103) were female. Most had 9-12 years of education (54/103, 52%) and belonged to the most vulnerable socioeconomic category according to the Social Registry of Households classification (67/103, 65%). Overall, both groups had similar baseline characteristics (*P*>.05; Multimedia Appendix 2).

There were no significant baseline differences between those participants randomized and those included in the per-protocol analysis (*P*>.05).

### Primary Outcomes

#### Within-Group Effects

Both the comparator and telehealth groups showed significant pre-post improvements across most oral health outcomes (Table 1).

**Table 1:** Baseline and postintervention scores by study group.

Variable	Comparator group (n=51)				Telehealth group (n=52)				*P* value^a^
	Pretest, mean (95% CI)	Posttest, mean (95% CI)	*P* value^b^	Pretest, mean (95% CI)		Posttest, mean (95% CI)	*P* value^b^		
Dental caries knowledge	9.6 (9.11-10.09)	10.1 (9.65-10.58)	.08	9.5 (8.96-10.03)		10.7 (10.29-11.12)	<.001	.046	
Periodontal disease knowledge	8.6 (7.67-9.54)	10.2 (9.89-10.65)	.002	8.0 (6.91-9.08)		10.5 (10.11-10.99)	<.001	.31	
Oral cancer knowledge	5.4 (4.25-6.56)	8.7 (8.04-9.48)	<.001	6.0 (4.87-7.12)		9.1 (8.35-9.91)	<.001	.59	
Total knowledge	23.6 (21.59-25.65)	29.1 (27.93-30.37)	<.001	23.5 (21.30-25.69)		30.4 (29.12-31.68)	<.001	.14	
Oral health attitudes	4.0 (3.59-4.44)	3.9 (3.55-4.36)	.80	3.9 (3.59-4.36)		4.9 (4.44-5.40)	.002	.002	
OHSES^c^	5.2 (4.65-5.93)	6.7 (6.51-6.89)	<.001	5.0 (4.54-5.53)		6.5 (6.25-6.74)	<.001	.44	

^a^Between-group comparisons at posttest (adjusted for baseline).

^b^Within-group comparisons (pre vs post).

^c^OHSES: Oral Health Self-Efficacy Scale.

Participants in the comparator group (face-to-face education; n=51) showed large effects in oral health knowledge (Cohen *d*=0.93, 95% CI 0.52-1.34; *P*<.001), oral cancer knowledge (*r*=0.63, 95% CI 0.36-0.90; *P*<.001), oral health self-efficacy (*r*=0.59, 95% CI 0.38-0.74; *P*<.001), and toothbrushing behavior (*r*=0.51, 95% CI 0.24-0.79; *P*<.001). Additionally, it had a moderate effect on periodontal disease knowledge (*r*=0.44, 95% CI 0.17- 0.71; *P*=.002). However, no significant changes were observed in attitudes (*P*=.76) or dental caries knowledge (*P*=.12).

In the telehealth education group (n=52), which combined face-to-face education with telehealth, improvements were even more marked across multiple domains. This group showed a large effect on dental caries knowledge (*r*=0.49, 95% CI 0.23-0.77; *P*<.001), attitudes toward oral health (*r*=0.42, 95% CI 0.14-0.69; *P*=.003), and periodontal disease knowledge (*r*=0.59, 95% CI 0.31-0.86; *P*<.001). Similar to the comparator group, the telehealth education group also exhibited large effects in oral health knowledge (Cohen *d*=1.07, 95% CI 0.66-1.48; *P*<.001), oral health self-efficacy (*r*=0.62, 95% CI 0.43-0.77; *P*<.001), toothbrushing behavior (*r*=0.54, 95% CI 0.26-0.81; *P*<.001), and oral cancer knowledge (*r*=0.60, 95% CI 0.33-0.87; *P*<.001).

**Figure 2:**
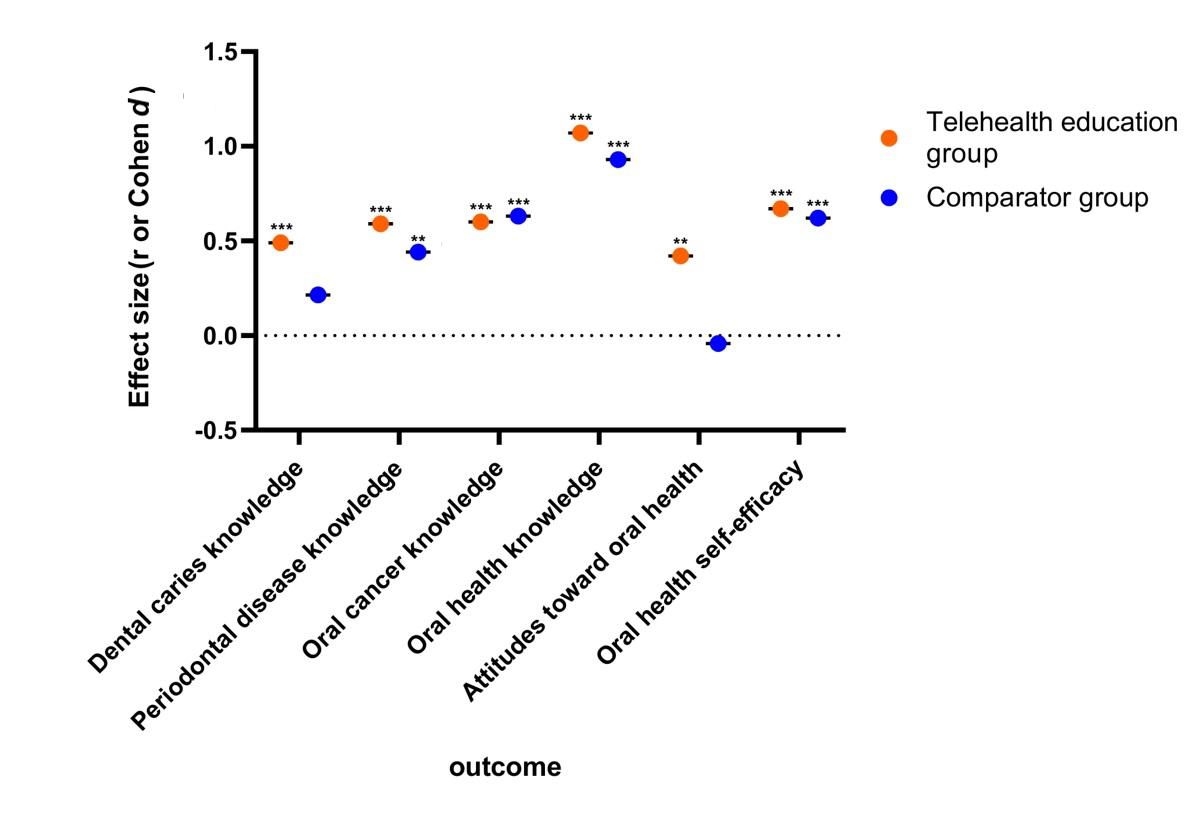
Effect sizes for preintervention to postintervention differences in oral health knowledge, attitudes toward oral health, and oral health self-efficacy, stratified by intervention group. **P<.01, ***P<.001.

Figure 2 visualizes the magnitude of effects across groups and outcomes, with asterisks indicating significance levels.

#### Detailed Changes in Knowledge Subdomains

##### Total Knowledge Score

Total knowledge scores were comparable at baseline (*P*=.93; Table 1). Following the intervention, both groups demonstrated substantial within-group improvements: the comparator group increased by 5.5 (95% CI 3.37-7.68) points (*P*<.001), and the telehealth group improved by 6.9 (95% CI 4.70-9.10) points (*P*<.001). Notably, no participant achieved the maximum possible score of 39 points.

##### Dental Caries Knowledge

Baseline knowledge of caries signs and symptoms was high across the sample (mean score 4.4 out of 6, SD 1.38), with no significant differences between groups (*P*=.12). The most frequently recognized signs included the presence of a cavity (92/103, 89%), and persistent pain (85/103, 82%), while tooth pain when consuming sweets was the least recognized (68/103, 66%).

**Figure 3:**
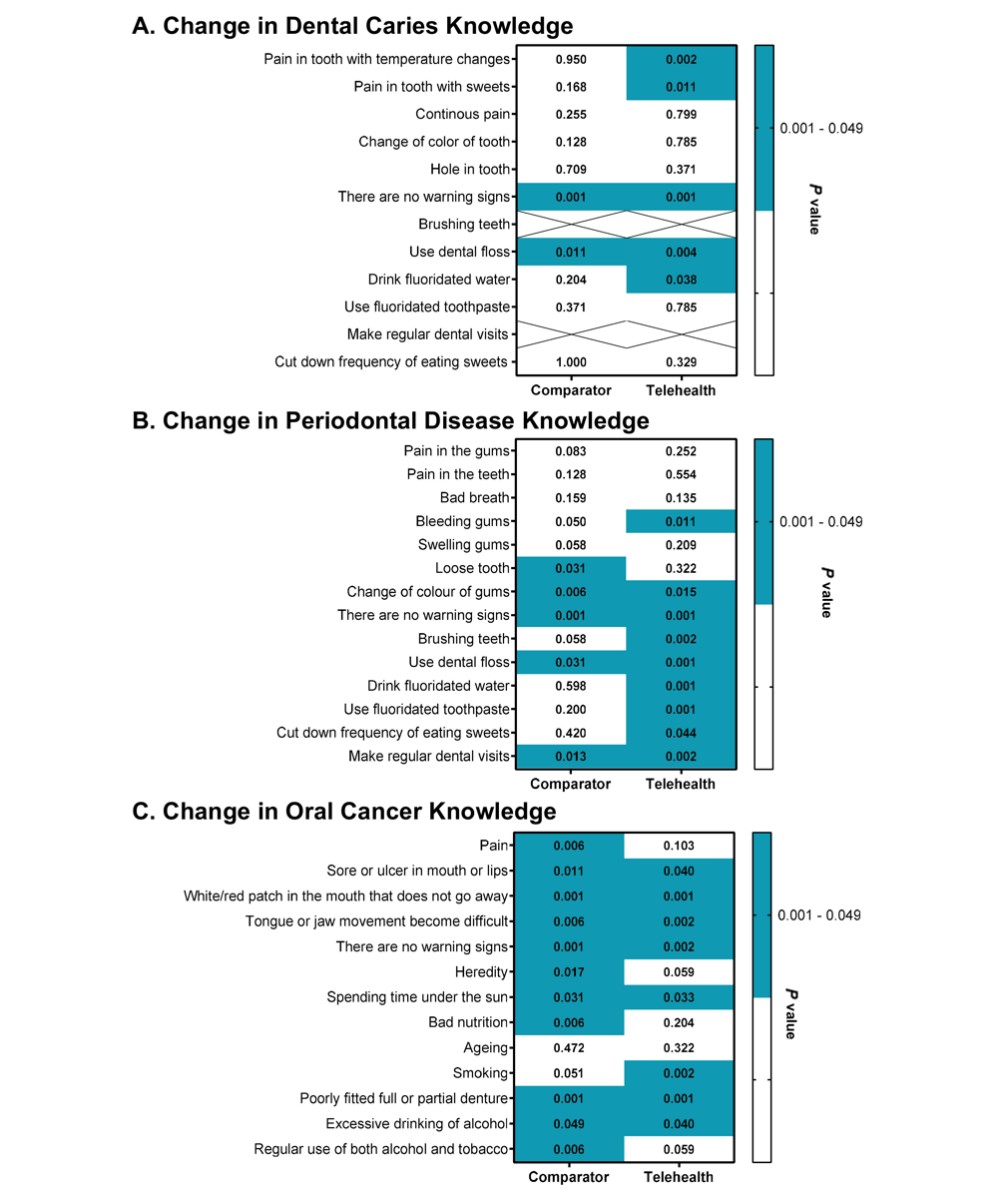
Preintervention to postintervention changes in participants’ knowledge related to dental caries, periodontal disease, and oral cancer, stratified by group. Statistically significant improvements are highlighted in blue.

Similarly, awareness of preventive measures was high overall (mean score 5.1 out of 6, SD 0.9), with no significant differences between intervention groups (comparator: mean 5.0, 95% CI 4.73-5.26; telehealth: mean 5.30, 95% CI 5.05-5.55; *P*=.09). However, the largest knowledge deficits were concentrated in 2 specific preventive measures, fluoride in water (61/103, 59%) and flossing (77/103, 79%). After the intervention, both items showed significant increases in the telehealth education group, reaching 83% (43/52; *P*=.03) and 100% (52/52; *P*=.004), respectively (Figure 3).

##### Periodontal Disease Knowledge

At baseline, the mean score for identifying signs and symptoms of periodontal disease was 5.5 (SD 2.6) out of 8 points. Scores were similar between groups (comparator: mean 5.6, 95% CI 4.99-6.37; telehealth: mean 5.5, 95% CI 4.70-6.33; *P*=.75). Recognition was below 80% for all signs and symptoms, except for gum inflammation (87/103, 85%). Notably, approximately 70% (72/103) of the overall sample believed that periodontal disease has no signs or symptoms.

Baseline recognition of preventive measures was generally low, mean 2.7 (SD 1.2) out of 5 points, without differences across groups (comparator: mean 2.9, 95% CI 2.54-3.29; telehealth: mean 2.48, 95% CI 2.15-2.80; *P*=.07). Most participants (77%-95%), held misconceptions about the role of fluoride in preventing periodontal disease, and these misconceptions persisted even after the intervention. However, the telehealth group demonstrated partial improvement, with a significant decrease specifically in the misconception associating fluoride in toothpaste with periodontitis prevention (*P*<.001; Figure 3).

Following the intervention, participants showed significant improvements in identifying signs, symptoms, and preventive measures (*P*<.001). These gains were particularly evident in the telehealth education group, which demonstrated significantly better recognition of preventive measures (comparator: mean 3.3, 95% CI 3.15-3.63; telehealth: mean 3.7, 95% CI 3.52-4.04; *P*=.01).

##### Oral Cancer Knowledge

Baseline knowledge of oral cancer signs, symptoms, and risk factors was low across the sample, with mean scores of 1.7 (SD 1.7) out of 5 and 3.8 (SD 2.8) out of 8, respectively. Overall, 69% (71/103) of participants were unable to identify signs or symptoms, and 24% (25/103) were unaware of risk factors. The most frequently recognized sign was a persistent oral ulcer or wound (53/103, 51%), and smoking was the most identified risk factor (74/103, 72%).

At pretest, there were no significant differences between groups in the identification of signs and symptoms (comparator: mean 1.8, 95% CI 1.29-2.31; telehealth: mean 1.7, 95% CI 1.26-2.27; *P*=.92) or in awareness of risk factors (comparator: mean 3.6, 95% CI 2.86-4.34; telehealth: mean 4.2, 95% CI 3.44-5.01; *P*=.24).

Following the intervention, both groups demonstrated a significant increase in the identification of signs, symptoms, and risk factors *(P*<.001). Posttest comparisons showed no significant between-group differences in identification (comparator: mean 3.5, 95% CI 3.15-3.90; telehealth: mean 3.3, 95% CI 2.98-3.78; *P*=.60) or in risk-factor awareness (comparator: mean 5.2, 95% CI 4.72-5.74; telehealth: mean 5.5, 95% CI 5.24-6.25; *P*=.057). Across the sample, 90% (93/103) of participants could identify at least 2 signs or symptoms of oral cancer, and 96% (99/103) recognized at least 2 risk factors. Nearly all individual indicators showed statistically significant improvement (*P*<.05; Figure 3).

#### Attitudes Toward Oral Health

No statistically significant differences in baseline attitude scores were observed between groups (comparator: mean 4.0, 95% CI 3.59-4.44; telehealth: mean 3.9, 95% CI 3.59-4.36; *P*=.89). Following the intervention, only the telehealth group demonstrated statistically significant improvement (mean difference 0.9, 95% CI 0.37-1.51, *P*=.002; Table 1). 

Notable changes were observed in the reduction of agreement with negative attitudes, with all improvements favoring the telehealth education group. Specifically, telehealth participants were less likely to agree with statements such as “As people become older, they can expect to lose their teeth” (*P*=.01), “Only people with teeth need to visit the dentist” (*P*=.01), and “Older adults must learn to live with the condition of their teeth and gums” (*P*=.02).

However, a highly prevalent negative baseline attitude—the belief that “Having dental problems is a normal part of aging” (87/103, 84%)—did not significantly change following the intervention in either group (*P*=.18).

#### Oral Health Self-Efficacy

At baseline, OHSES scores were comparable between groups (comparator: mean 5.2, 95% CI 4.65-5.93; telehealth: mean 5.0, 95% CI 4.54-5.53; *P*=.52). Most items were rated positively (>50%), except confidence in using dental floss (49/103, 47%), which was significantly lower in the telehealth group (*P*=.03)

Following the intervention, total OHSES scores increased significantly (*P*<.001) across all individual items (*P*<.01) and intervention groups (*P*<.001; Table 1). No between-group posttest differences were detected (*P*=.44). The proportion of participants achieving the maximum score rose from 32% (33/103) to 75.7% (78/103), and all items exceeded 80% at follow-up.

#### Between-Group Comparisons

When comparing the postintervention scores adjusted for baseline (Table 1), the telehealth group was significantly superior to the comparator group in 2 primary outcomes. Specifically, at 6 weeks, participants receiving the telehealth intervention (52/103) were significantly more likely than those in the comparator group (51/103) to demonstrate improved knowledge about dental caries (*r*=0.21, 95% CI 0.11-0.3; *P*=.03) and more positive attitudes toward oral health (*r*=0.28, 95% CI 0.18-0.38; *P*=.004), corresponding to small and moderate effect sizes, respectively. For all other primary outcomes, no significant between-group differences were detected at the posttest assessment (*P*>.05; Table 1).

### Secondary Outcomes

#### Sensitivity Analysis: ITT

The ITT analysis was conducted to assess the effect of the intervention on the full randomized sample (n=120). Missing data were handled using multiple imputation, where all prespecified sociodemographic, clinical, pretest, posttest, and group assignment variables were included in the imputed model.

Between-group analysis showed that the effect on posttest dental caries knowledge was no longer statistically significant (B=0.60, SE 0.32, 95% CI -0.03-1.23; *P*=.06), whereas the telehealth advantage for oral health attitudes remained significant (B=0.91, SE 0.29, 95% CI 0.34-1.48; *P*=.002; Table 2).

**Table 2:** Intention-to-treat analysis results by group using multiple imputation.

Variable	Comparator group (n=59)				Telehealth group (n=61)				*P* value^a^
	Pretest, mean (95% CI)	Posttest, mean (95% CI)	*P* value^b^	Pretest, mean (95% CI)		Posttest, mean (95% CI)	*P* value^b^		
							
Dental caries knowledge	9.6 (9.11-10.09)	10.1 (9.66-10.60)	.05	9.5 (8.96-10.03)		10.7 (10.33-11.12)	<.001	.06	
Periodontal disease knowledge	8.6 (7.67-9.54)	10.2 (9.90-10.66)	<.001	8.0 (6.91-9.08)		10.5 (10.16-10.98)	<.001	.30	
Oral cancer knowledge	5.4 (4.25-6.56)	9.0 (8.29-9.75)	<.001	6.0 (4.87-7.12)		9.1 (8.42-9.81)	<.001	.75	
Total knowledge	23.6 (21.59-25.65)	29.3 (27.95-30.69)	<.001	23.5 (21.30-25.69)		30.3 (28.96-31.66)	<.001	.11	
Oral health attitudes	4.0 (3.59-4.44)	3.9 (3.58-4.38)	.87	3.9 (3.59-4.36)		4.8 (4.45-5.33)	<.001	.002	
OHSES^c^	5.2 (4.65-5.93)	6.6 (6.45-6.92)	<.001	5.0 (4.54-5.53)		6.4 (6.27-6.70)	<.001	.59	

^a^Between-group comparisons at posttest (adjusted for baseline).

^b^Within-group comparisons (pre vs post).

^c^OHSES: Oral Health Self-Efficacy Scale.

To assess the stability of the primary conclusion regarding attitudes against potential MNAR mechanisms, a Worst-Case Reasonable Scenario sensitivity analysis was conducted. Between-group difference in oral health attitudes remained significant under the MNAR mechanisms assumption (*P*=.02). This finding confirms the stability of the telehealth intervention’s effect on attitudes, despite potential attrition bias.

#### Subgroup Analyses by Place of Residence

Secondary exploratory analyses examined response patterns by place of residence (rural vs urban). Rural participants accounted for 50% (60/120) of the randomized sample, with no significant differences between intervention groups (*P*=.85) or in the final analyzed sample (*P*=.27).

At baseline, rural participants showed greater vulnerability, including differences in smartphone use (*P*=.002), higher multimorbidity (*P*=.01), and fewer number of teeth present (rural: mean 16.24, SD 7.15; urban: mean 19.20, SD 7.16; *P*=.03), and consistently lower knowledge across all domains (all *P*<.05; Multimedia Appendix 3).

**Figure 4:**
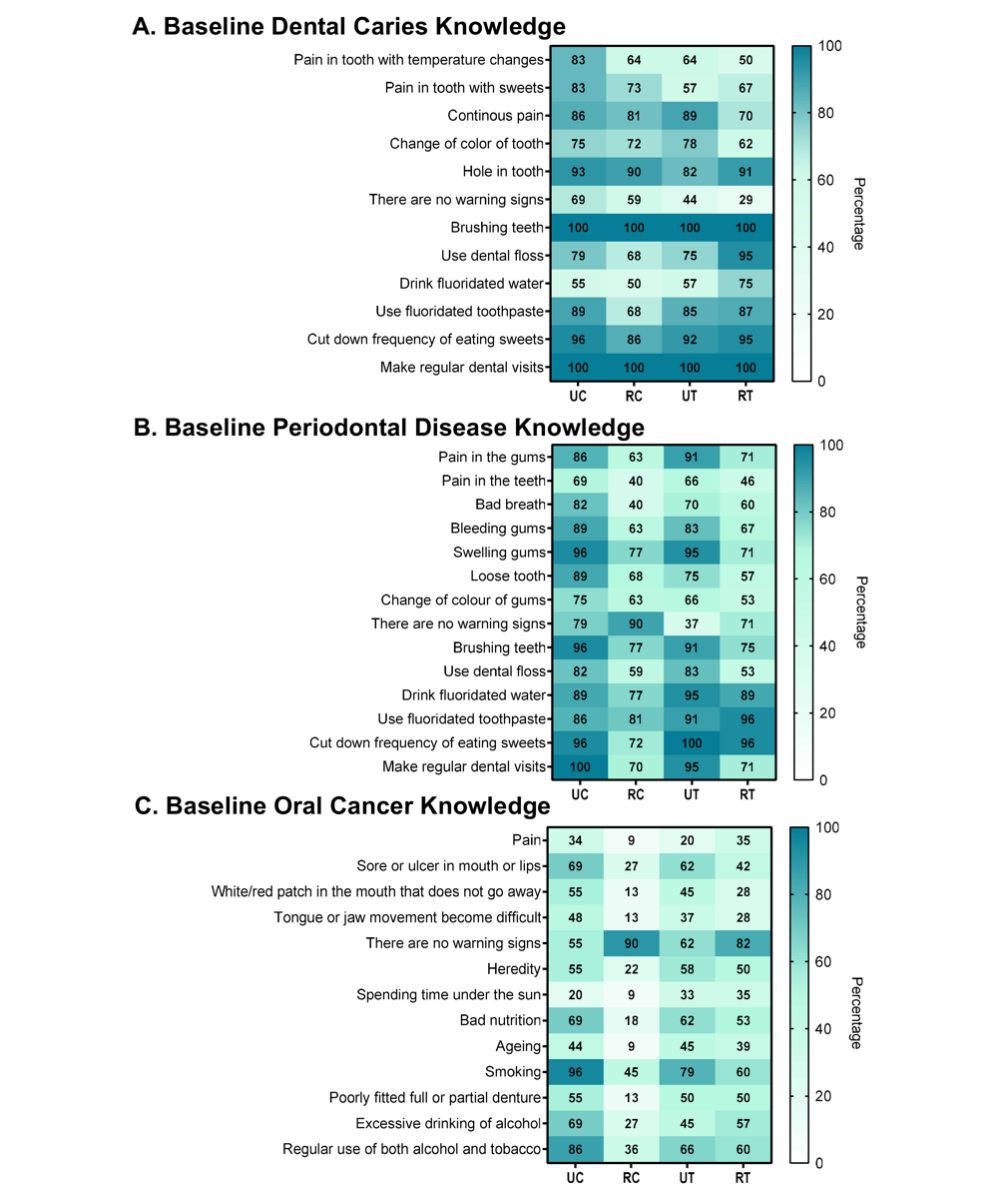
Heatmap of baseline oral health knowledge related to dental caries, periodontal disease, and oral cancer, expressed as the percentage of participants responding “yes” to each question. Results are stratified by group and place of residence. The color gradient ranges from white (lower percentage) to blue (higher percentage) of affirmative responses. RC: rural comparator; RT: rural telehealth education; UC: urban comparator; UT: urban telehealth education.

Urban participants demonstrated significantly higher baseline knowledge of dental caries (*P*=.04), periodontal disease (*P*<.001), and oral cancer knowledge (*P*<.001). Item-level percentages illustrating these baseline disparities are presented in Figure 4, while Table 3 summarizes the corresponding pre-post differences by group and place of residence. At baseline, a substantial proportion of rural participants were unable to identify key signs of periodontal disease (12/50, 24% rural vs 1/53, 1.8% urban; *P*=.01) or oral cancer; specifically 86% (43/50) of rural participants could not identify any signs or symptoms of oral cancer, and 40% (20/50) were unaware of risk factors, compared with 9% (5/53) among urban participants (*P*=.003).

**Table 3:** Comparison of scores in oral health knowledge, attitudes, self-efficacy, and self-reported oral hygiene behaviors before and after intervention, by group and place of residence.

Variable, group, and place of residence				Pretest		Posttest		*P* value
**Dental caries knowledge, mean (95% CI)**								
	**Telehealth education**							
		Rural (n=28)	9.3 (8.53-10.17)		10.9 (10.28-11.50)		<.001^a^	
		Urban (n=24)	9.7 (8.93-10.39)		10.5 (9.91-11.08)		.08^a^	
	**Comparator**							
		Rural (n=22)	8.9 (8.03-9.87)		10.0 (9.31-10.77)		.04^a^	
		Urban (n=29)	10.1 (9.62-10.58)		10.2 (9.53-10.80)		.85^a^	
**Periodontal disease knowledge, mean (95% CI)**								
	**Telehealth education**							
		Rural (n=28)	7.1 (5.38-8.97)		10.8 (10.17-11.46)		<.001^a^	
		Urban (n=24)	8.9 (7.86-10.04)		10.2 (9.63-10.86)		.02^a^	
	**Comparator**							
		Rural (n=22)	7.1 (5.19-8.98)		10.5 (9.86-11.13)		.004^a^	
		Urban (n=29)	9.7 (9.12-10.39)		10.1 (9.62-10.58)		.20^a^	
**Oral cancer knowledge, mean (95% CI)**								
	**Telehealth education**							
		Rural (n=28)	5.6 (3.96-7.24)		10.2 (9.12-11.23)		<.001^a^	
		Urban (n=24)	6.4 (4.83-8.08)		7.9 (6.89-8.93)		.19^a^	
	**Comparator**							
		Rural (n=22)	2.5 (1.02-4.06)		8.2 (6.95-9.50)		<.001^a^	
		Urban (n=29)	7.5 (6.38-8.78)		9.2 (8.30-10.04)		.02^a^	
**Total knowledge, mean (95% CI)**								
	**Telehealth education**							
		Rural (n=28)	22.1 (18.69-25.59)		31.9 (30.01-33.77)		<.001^b^	
		Urban (n=24)	25.1 (22.40-27.75)		28.7 (27.12-30.20)		.02^b^	
	**Comparator**							
		Rural (n=22)	18.5 (15.46-21.71)		28.8 (26.87-30.67)		<.001^b^	
		Urban (n=29)	27.4 (25.70-29.18)		29.4 (27.76-31.13)		.05^b^	
**Oral health attitudes, mean (95% CI)**								
	**Telehealth education**							
		Rural (n=28)	3.8 (3.31-4.40)		5.2 (4.53-5.82)		.004^a^	
		Urban (n=24)	4.1 (3.55-4.69)		4.6 (3.87-5.37)		.25^a^	
	**Comparator**							
		Rural (n=22)	3.7 (2.97-4.38)		3.6 (3.16-4.01)		.79^a^	
		Urban (n=29)	4.3 (3.73-4.81)		4.2 (3.60-4.88)		.85^a^	
**OHSES** ^c^ **, mean (95% CI)**								
	**Telehealth education**							
		Rural (n=28)	5.3 (4.80-5.83)		6.5 (6.24-6.89)		<.001^a^	
		Urban (n=24)	4.7 (3.78-5.62)		6.4 (6.00-6.82)		.002^a^	
	**Comparator**							
		Rural (n=22)	5.1 (4.01-6.25)		6.8 (6.65-7.07)		.004	
		Urban (n=29)	5.4 (4.62-6.20)		6.5 (6.28-6.88)		.002^a^	
**Toothbrushing frequency, mean (95% CI)**								
	**Telehealth education**							
		Rural (n=28)	2.3 (2.06-2.58)		2.9 (2.69-3.16)		<.001^a^	
		Urban (n=24)	2.5 (2.08-2.91)		2.9 (2.56-3.18)		.03^a^	
	**Comparator**							
		Rural (n=22)	2.5 (2.24-2.84)		3.0 (2.69-3.39)		.008^a^	
		Urban (n=29)	2.6 (2.36-2.94)		3.0 (2.64-3.42)		.01^a^	
**Flossing, n (%)**								
	**Telehealth education**							
		Rural (n=28)	8 (29)		21 (75)		<.001^d^	
		Urban (n=24)	12 (50)		15 (62)		.37^d^	
	**Comparator**							
		Rural (n=22)	8 (36)		11 (50)		.45^d^	
		Urban (n=29)	10 (34)		17 (59)		.06^d^	

^a^Wilcoxon signed-rank test.

^b^Paired *t* test.

^c^OHSES: Oral Health Self-Efficacy Scale.

^d^McNemar test.

Across both study arms, rural participants exhibited larger within-group pre-post improvements than urban participants. In the telehealth group, rural participants showed significant gains across all knowledge domains (all *P*<.001), OHSES (*P*<.001), and were the only subgroup to show significant improvements in attitudes (*P*=.004) and flossing (*P*<.001). Urban participants improved in fewer outcomes (Table 3). A similar pattern was observed in the comparator group, where rural participants improved across all knowledge components and OHSES, whereas several pre-post comparisons among urban participants were nonsignificant. Overall, improvements were more consistent and pronounced among rural subgroups, particularly in the telehealth arm. However, these findings are exploratory and reflect within-group changes only; limited sample sizes across the 4 intervention-residence subgroups do not allow for formal comparative analyses.

#### Self-Reported Oral Hygiene Behaviors

At baseline, nearly all participants reported brushing their teeth regularly (102/103, 99%), with 54% (56/103) brushing once or twice daily. At baseline, there were no significant differences between groups (comparator: mean 2.6, 95% CI 2.40-2.81; telehealth: mean 2.4, 95% CI 2.17-2.63; *P*=.18). Following the intervention, 75% (77/103) reported brushing 3 or more times per day, with significant within-group increases (both *P*<.001). However, baseline-adjusted posttest comparisons showed no significant differences between groups (comparator: mean 3.0, 95% CI 2.78-3.29; telehealth: mean 2.9, 95% CI 2.71-3.08; *P*=.90). The change in toothbrushing frequency showed a weak yet significant correlation with perceived self-efficacy in thoroughly brushing all tooth surfaces (*r*=0.196; *P*=.04).

At baseline, 63% (65/103) of the participants reported not using dental floss, with no significant differences between groups (*P*=.73). Pretest flossing behavior was significantly associated with baseline attitudes toward oral health (*P*=.01), oral health self-efficacy (*P*=.04), educational level (*P*=.007), and number of teeth present (*P*=.01). A moderate positive correlation was found between baseline flossing behavior and baseline flossing self-efficacy (*r*=0.41; *P*<.001), while postintervention flossing demonstrated a weaker yet significant correlation (*r*=0.29; *P*=.003).

The frequency of self-reported flossing significantly increased following the intervention (38/103, 36.9% vs 64/103, 62.1%; *P*<.001). Participants in the telehealth group showed a higher likelihood of reporting flossing compared to those in the comparator group (risk ratio=1.31, 95% CI 0.96-1.78), corresponding to an absolute increase in flossing prevalence of 16.7% (risk difference=0.16, 95% CI –0.02 to 0.35); however, this between-group difference was not statistically significant (*P*=.08).

#### Smartphone Usage and Telehealth Education Acceptability

Among those assessed for eligibility, 25% (105/420) reported not using WhatsApp (Figure 1), with this pattern more pronounced in rural areas. No significant differences in WhatsApp use were found between study groups (*P*=.86). However, rural residents were significantly less likely to use online search tools (*P*=.04), other social media apps (*P*=.04), or email (*P*<.001). Advanced smartphone use—defined as use of WhatsApp, online search, social media, and email—was reported by 45% (24/53) of urban participants (Multimedia Appendix 3). Limited smartphone users (WhatsApp only) were significantly more likely to have ≤8 years of formal education and live in rural areas (*P*=.008).

Of the eligible participants, 63.1% (120/190) agreed to enroll in the intervention (willingness to participate; Figure 1). Overall, the study experienced a 14.2% (17/120) attrition rate. At 6 weeks, dropout rates did not differ significantly between the telehealth education group (9/61, 15%) and the comparator group (8/59, 13%; *P*=.85).

Most participants of the telehealth education group (48/52, 92%) stated they would recommend the use of telehealth education videos, indicating a positive attitude toward the intervention. Recommendation rates did not significantly differ by place of residence (*P*=.84), educational level (*P*=.29), or smartphone usage (*P*=.27). Perceived effectiveness of the instructional videos was high, with all participants finding the videos on dental hygiene, prevention of dental caries, and oral cancer prevention useful (52/52, 100%), and 90% (47/52) finding the periodontal disease prevention video useful.

#### Cluster Analysis of Demographic- and Intervention-Related Variables in the Telehealth Education Group

The analysis identified 2 main dimensions, explaining 56.5% of the total variance. Dimension 1 primarily captured the relationships between smartphone use, place of residence, and changes in knowledge and attitude scores, while dimension 2 reflected educational level and changes in oral health self-efficacy.

**Figure 5:**
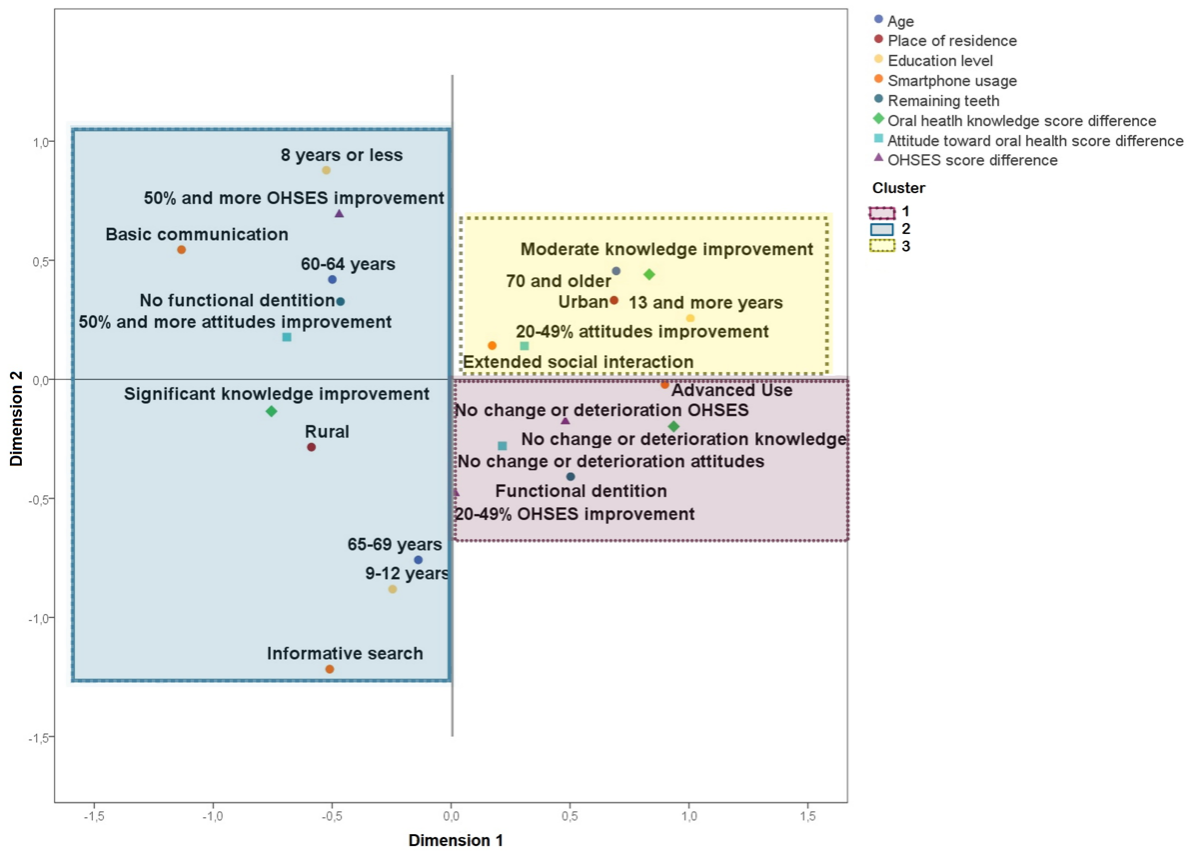
Subgroup profiles within the telehealth education group were identified using multiple correspondence analysis followed by k-means clustering. Each cluster (cluster 1: pink, square-dotted line; cluster 2: blue, solid line; cluster 3: yellow, round-dotted line) corresponds to distinct patterns of sociodemographic and clinical characteristics, including age, place of residence, education level, smartphone use, and number of remaining teeth. These clusters show differential improvements in oral health knowledge, attitudes, and Oral Health Self-Efficacy Scale scores.

Subsequent k-means clustering identified 3 distinct participant profiles (Figure 5):

- Cluster 1: participants who derived little to no benefit from the intervention. This cluster included individuals with functional dentition (12/22, 86%; *χ*^2^_2_=11.65; *P*=.003; post hoc Dwass-Steel-Critchlow-Fligner: C1>C2,*P*=.002; C1>C3, *P*=.04), who showed minimal or negative changes in knowledge, attitudes, and oral health self-efficacy.
- Cluster 2: participants who benefited the most, with most members showing high improvement (≥50%) in oral health knowledge (20/22, 91%; *χ*^2^_2_=23.24; *P*<.001; post hoc Dwass-Steel-Critchlow-Fligner: C2>C1, *P*=.01; C2>C3, *P*<.001), particularly in oral cancer knowledge (13/22, 59%; *χ*^2^_2_=17.56; *P*<.001; post hoc Dwass-Steel-Critchlow-Fligner: C2>C1, *P*=.01; C2>C3, *P*=.04), and in self-efficacy (12/22, 54%;*χ*^2^_2_=9.33; *P*=.009; post hoc Dwass-Steel-Critchlow-Fligner: C2>C1, *P*=.01; C2>C3, *P*=.04) This cluster was characterized by rural residence (16/22, 73%; *χ*^2^_2_=8.57; *P*=.01; post hoc Dwass-Steel-Critchlow-Fligner: C2>C3, *P*=.02), and limited smartphone use (11/22, 50%; *χ*^2^_2_=15.60; *P*<.001; post hoc Dwass-Steel-Critchlow-Fligner: C2>C3, *P*<.001).
- Cluster 3: participants with moderate improvements. This group consisted mainly of urban adults (12/16, 75%; *P*=.01) aged ≥70 years (13/22, 81.3%; *χ*^2^_2_=0.25.00; *P*<.001; post hoc Dwass-Steel-Critchlow-Fligner: C3>C1, *P*<.001; C3>C2, *P*<.001) and smartphone use oriented toward extended social interaction (9/16, 56.3%; *P*<.001).

### Harms and Unintended Events

No clinical harms or adverse health-related events were observed. However, one unintended operational issue arose in the telehealth group: 5 (8.2%) participants of the 61 assigned to telehealth—despite initially reporting confidence using WhatsApp—were unable to access or view the educational videos, 4 of whom lived in rural areas. This usability-related limitation led to their exclusion from the per-protocol analysis but did not pose any health risks.

## Discussion

### Principal Findings

This randomized trial tested a WhatsApp‑supported telehealth reinforcement strategy among community-dwelling older adults in the La Araucanía Region of Chile. Both study arms demonstrated significant improvements in oral health knowledge and self-efficacy. Between-group differences favored telehealth only for attitudes toward oral health and dental caries knowledge in the primary per-protocol analysis, with attitudes showing the strongest effect. No significant differences between groups were identified for total knowledge, periodontal disease recognition, or oral cancer knowledge. These findings underscore the potential of mHealth interventions in addressing disparities in preventive care, aligning with World Health Organization recommendations [88].

Intention-to-treat sensitivity analysis attenuated the between-group difference in dental caries knowledge, while superiority for attitudes remained. This outcome suggests that the added benefit of telehealth reinforcement, while present, was modest and more evident among participants with full adherence to the intervention. A plausible explanation is the high effectiveness of the face-to-face education delivered to the comparator group, which produced substantial baseline improvements, thereby reducing the margin for further short-term gains. Similar patterns have been reported in prior education trials, where reinforcement strategies yielded clearer benefits over longer follow-up periods rather than immediately post intervention [15].

From an SCT perspective, learning and behavior change are supported by reinforcement and repeated opportunities to observe, rehearse, and apply learned behaviors in supportive environments [22,25]. While face-to-face education appeared sufficient for most knowledge domains, educational research indicates that single-session interventions are vulnerable to declining retention over time in the absence of reinforcement [89,90]. In this study, telehealth operated as a structured extension of the in-person education, allowing repeated exposure to key concepts and practical application through a familiar digital platform—an approach aligned with older adults’ preference for reliable postconsultation resources [46,52,53]. Although absolute knowledge gains were slightly larger in the telehealth group, no statistically significant between-group differences were observed. Notably, the magnitude of improvement observed in the telehealth arm falls at the upper range reported in prior digital interventions for older adults (3.1-5.4 points) [31,45,91], although comparisons should be interpreted cautiously given heterogeneity in study designs and outcome measures. Taken together, this combined mechanism may help explain the between-group gains observed in specific knowledge domains—particularly dental caries and periodontal prevention—among fully adherent participants.

Beyond knowledge acquisition, effective preventive behavior change requires additional mechanisms, such as self-efficacy [92]. Prior research suggests that gains in knowledge can positively influence attitudes and self-efficacy, supporting the adoption of healthier behaviors, and, over time, improvements in oral-health related quality of life [16]. Both groups demonstrated substantial improvements in self-efficacy, which were positively associated with self-reported improvements in hygiene behaviors. Exploratory subgroup analysis further suggested that rural participants receiving telehealth reinforcement experienced additional behavioral gains, specifically increased flossing. Although exploratory, this pattern is consistent with SCT-based mechanisms—such as observational learning (video-based demonstrations), verbal persuasion (via simple and supportive messaging), environmental facilitation (home-based, flexible access to content), and repetition for mastery (allowing participants to review and practice skills introduced during the face-to-face session) [22,25]. Together, these elements may have strengthened self-regulation, reinforced self-efficacy, and supported the internalization of health recommendations, thereby promoting skill development and behavior adoption [21].

The improvement in prevention-oriented attitudes may reflect changes in outcome expectancies, a core SCT construct referring to beliefs about the anticipated benefits of a behavior [22,25]. Repeated, self-paced engagement with preventive content may have strengthened participants’ expectations that preventive oral health behaviors are effective and worthwhile, thereby supporting more positive attitudes toward oral health in older age. However, the persistence of fatalistic beliefs—such as viewing oral deterioration as an inevitable part of aging—highlights the resistance of deeply-rooted social narratives, particularly in socioeconomically vulnerable groups [93]. Addressing normative beliefs may require culturally sensitive messaging, accessible education, and stronger community engagement strategies to build collective efficacy and shift these deeply rooted perceptions [94].

Exploratory subgroup analyses suggested that the intervention may have helped reduce baseline knowledge disparities among rural participants. These differences aligned with underlying oral health inequalities, as rural participants had fewer teeth on average—an indicator commonly associated with limited access to preventive services and delayed diagnosis among higher-risk populations [2,95]. Findings from the multiple correspondence analysis and k-means clustering further refined this interpretation. Although the cluster showing the greatest improvements was predominantly rural, another cluster with mixed urban-rural composition and higher rates of functional dentition displayed limited responsiveness. This pattern suggests that baseline preventive need, proxied by dentition status, may play a more influential role than residence alone in shaping intervention response. Accordingly, delivering educational content through accessible digital platforms may be particularly beneficial for older adults with greater unmet preventive needs—many of whom reside in rural settings [95]—by helping mitigate structural barriers in high-need populations. This approach may be especially relevant in settings with limited access to oral health care and quality educational resources, while also representing a potentially cost-effective strategy to narrow rural-urban disparities in oral health promotion [12,95,96].

The study also highlights persistent challenges related to the “gray digital divide” [40], as a notable proportion of otherwise eligible participants were excluded due to limited WhatsApp skills. To address this, the intervention incorporated inclusive design principles by using familiar social media tools, providing basic digital guidance, and videos tailored and validated for older adults with simple language, visual modeling, and accessibility features such as large fonts, high-contrast images, clear audio, appropriate pacing, and mobile-friendly formats. Despite these actions, some telehealth participants still faced access difficulties. Although this represents a minor unintended event rather than a clinical harm, it underscores how digital competencies—unequally distributed, especially in rural areas—can limit engagement [97,98]. Combining social media use with digital literacy training can enhance eHealth engagement in older adults [99,100].

Overall, the reinforcement was well received and perceived as useful, with high willingness to recommend the intervention, regardless of sociodemographic characteristics. Incorporating older adults’ perspectives in co-design may further improve relevance, usability, and equity of digital health tools [53,101]. Recent qualitative research using the Unified Theory of Acceptance and Use of Technology framework highlights the importance of understanding older adults’ intentions, attitudes, and concerns to enhance telehealth uptake [102]. The TEGO enabled continuous monitoring and identification of subgroups—such as participants with functional dentition or higher baseline knowledge—who may benefit from tailored content, aligning with prior teledentistry recommendations [43].

From a public health perspective, these findings support the use of smartphone-based telehealth reinforcement as a complementary component of in-person oral health promotion programs, particularly in settings facing structural barriers to preventive care. Although effects were modest, incremental gains—especially among rural older adults—suggest that targeted telehealth interventions can improve access and reinforce learning where traditional services are limited, potentially preventing more costly oral and systemic complications over time [103-106]. Overall, telehealth reinforcement appeared feasible, acceptable, and safe; however, its impact was shaped by digital access, literacy, place of residence, and oral health status, which may limit its universal applicability.

Evidence on the cost-effectiveness of telehealth interventions in aging populations remains limited [107,108]. While economic evaluation was beyond the scope of this trial, findings highlight the importance of context-specific cost analyses, particularly for older adults with heterogeneous needs and patterns of health care use. Existing evidence indicates mixed results, with simpler mHealth approaches showing some benefit, unlike more complex smartphone-based apps [109]. Notably, cost-effectiveness varies across populations and settings, with greater savings reported in rural contexts, and more favorable outcomes for in-person care in some urban minority groups [110]. These insights underscore the need for tailored economic evaluations that prioritize high‑need settings, define resource use clearly, use appropriate comparators, and adopt a societal perspective to capture both direct and indirect costs and benefits [111].

Scaling up WhatsApp-based or similar mHealth interventions in primary care will require investment in digital literacy training for health care professionals, integration of mobile-compatible tools into routine workflow, and strengthening technological infrastructure through reliable internet connectivity (eg, satellite or mobile broadband) and ensuring device availability, especially in rural areas [112,113]. Policy support is essential to promote adoption among patients, caregivers, and health teams, recognizing the role of mHealth in education, prevention, and continuity of care [114]. Moreover, safeguarding data privacy and controlling misinformation remain critical, as highlighted by recent qualitative evidence from health system decision-makers emphasizing coordinated policies across governments, clinicians, digital platforms, and the public [115].

### Limitations

While the study demonstrates promising results, it is important to recognize its limitations when interpreting the findings. First, the short follow-up period precluded assessment of the sustainability of changes in knowledge, attitudes, and self-efficacy. Second, although the intervention was guided by SCT, only self-efficacy was directly measured, whereas other constructs were embedded within the intervention design and assessed indirectly through participants’ engagement, knowledge acquisition, attitudes, and behavior change outcomes. Engagement with the educational videos was not systematically monitored, limiting evaluation of dose-response relationships or specific mechanisms of change. The absence of qualitative data—such as interviews or open-ended feedback—further restricted insight into participants’ subjective experiences and perceived usefulness of the intervention.

Self-reported measures were limited to secondary behavioral outcomes and may be subject to social desirability bias, warranting cautious interpretation. The relatively small sample size reduced statistical power for subgroup analyses and limited generalizability across geographic settings and levels of smartphone use. Subgroup findings, including differences by place of residence, were observational and should be interpreted as descriptive rather than causal. Finally, selection bias may have occurred at the eligibility stage due to unequal access to technology or differential motivation to participate, potentially limiting applicability to populations with lower digital proficiency.

### Implications and Future Directions

Despite these limitations, this study contributes to oral health promotion for older adults in underserved regions by demonstrating a structured application of a theory-informed intervention design. While individual components are not novel [17,18,21,31,43,116], their integration into a coherent strategy explicitly informed by SCT across face-to-face and telehealth delivery provides a concrete example of theory-informed intervention design, addressing documented gaps in explicit theory operationalization in oral health interventions targeting older adults [18,117,118]. Unlike most prior m-oral health studies, which have focused predominantly on urban populations [31,43-45,91,119], this study evaluates a geographically balanced rural-urban sample and offers suggestive evidence of differential intervention response across settings, a perspective rarely examined in previous studies. Overall, the findings indicate that theory-informed telehealth reinforcement may help address service gaps in underserved settings. The intervention demonstrated consistently high acceptability across multiple indicators, together with the use of accessible digital platforms—aligned with the digital context of older adults [50,51]—supporting its feasibility and suggesting potential for scalable, context-aligned implementation in resource-limited health systems [95,96]. By generating evidence in a local context, this study offers insights relevant to context-specific policy development and contributes to the growing literature on digital health for aging populations.

Looking ahead, future research should build upon these findings by using larger and more diverse samples, extending follow-up periods, and including clinical outcomes. Given the improvements observed, particularly among rural older adults, future studies should also explore how tailoring interventions to participants’ digital literacy, sociodemographic profiles, or baseline health status may enhance outcomes. Incorporating qualitative methods to capture participants‘ experiences, alongside in-depth analysis of their interaction with digital content (eg, process measures), will be essential to better understand the mechanisms underlying behavioral change. Furthermore, emerging technologies—such as artificial intelligence, gamification, or virtual reality—offer opportunities to enhance engagement, but must be coupled with digital literacy strategies to ensure equitable adoption.

### Conclusions

This study highlights the effectiveness of both face-to-face education and telehealth reinforcement, each grounded in SCT, in improving key oral health outcomes among older adults. Per-protocol analyses showed modest benefits of telehealth in specific domains—caries knowledge and attitudes—whereas ITT analyses confirmed significance only for attitudes. Exploratory subgroup findings suggest that individuals from rural settings and those with greater baseline preventive needs may derive added benefits, though these patterns should be interpreted cautiously. The acceptability of the telehealth approach further supports the feasibility of m-oral health and social media as a scalable strategy. Future research should examine long-term effects, impact on clinical outcomes, and explore adaptation for diverse populations and settings, ensuring their relevance and cost-effectiveness for broader implementation.

By examining how telehealth can reinforce high-quality face-to-face promotion, this study adds evidence supporting digital health strategies to promote oral health, particularly among vulnerable populations, and underscores their potential to advance healthy aging and equitable access to preventive care, encouraging further work to refine and expand this approach.

## Data Availability

The datasets generated or analyzed during this study are available from the corresponding author on reasonable request.

## Appendix

Multimedia Appendix 1 CONSORT-eHEALTH checklist (V 1.6.1).

Multimedia Appendix 2 Baseline sociodemographic, clinical, and oral health characteristics of participants by intervention group. Values are presented as mean (standard deviation) or percentage (number).

Multimedia Appendix 3 Baseline sociodemographic, clinical, and oral health characteristics of participants by intervention group and place of residence. Values are presented as mean (SD) or percentage (number). P values correspond to comparisons across the 4 subgroups (rural telehealth education, urban telehealth education, rural comparator, and urban comparator). The Kruskal-Wallis test was used for continuous variables.

## Abbreviations

ANCOVA: analysis of covariance
CONSORT: Consolidated Standards of Reporting Trials
CONSORT-EHEALTH: Consolidated Standards of Reporting Trials of Electronic and Mobile Applications and Online Telehealth
ICT: information and communications technology
I-CVI: Item Content Validity Index
ITT: intention-to-treat
mHealth: mobile health
MNAR: missing not at random
m-oral health: mobile oral health
OHSES: Oral Health Self-Efficacy Scale
SCT: social cognitive theory
TEGO: Geriatric Dental Specialties Tele-platform
