# Supplementary material for: WhatsApp-Supported Teledentistry to Reinforce Oral Health Promotion Among Older Adults Residing in Rural and Urban Areas: Randomized Controlled Trial
Source: JMIR Mhealth Uhealth. 2026 May 15;14:e71251. doi: 10.2196/71251 (PMC13221623; doi:10.2196/71251)
Supplement: Multimedia Appendix 1 [file mhealth_v14i1e71251_app1.pdf]

CONSORT-EHEALTH (V 1.6.1) - Submission/Publication Form

The CONSORT-EHEALTH checklist is intended for authors of randomized trials evaluating web-based and Internet-based applications/interventions, including mobile interventions, electronic games (incl multiplayer games), social media, certain telehealth applications, and other interactive and/or networked electronic applications. Some of the items (e.g. all subitems under item 5 - description of the intervention) may also be applicable for other study designs.

The goal of the CONSORT EHEALTH checklist and guideline is to be
a) a guide for reporting for authors of RCTs,
b) to form a basis for appraisal of an ehealth trial (in terms of validity)

CONSORT-EHEALTH items/subitems are MANDATORY reporting items for studies published in the Journal of Medical Internet Research and other journals / scientific societies endorsing the checklist.

Items numbered 1., 2., 3., 4a., 4b etc are original CONSORT or CONSORT-NPT (non-pharmacologic treatment) items.
Items with Roman numerals (i., ii, iii, iv etc.) are CONSORT-EHEALTH extensions/clarifications.

As the CONSORT-EHEALTH checklist is still considered in a formative stage, we would ask that you also RATE ON A SCALE OF 1-5 how important/useful you feel each item is FOR THE PURPOSE OF THE CHECKLIST and reporting guideline (optional).

Mandatory reporting items are marked with a red *.
In the textboxes, either copy & paste the relevant sections from your manuscript into this form - please include any quotes from your manuscript in QUOTATION MARKS,
or answer directly by providing additional information not in the manuscript, or elaborating on why the item was not relevant for this study.

YOUR ANSWERS WILL BE PUBLISHED AS A SUPPLEMENTARY FILE TO YOUR PUBLICATION IN JMIR AND ARE CONSIDERED PART OF YOUR PUBLICATION (IF ACCEPTED).
Please fill in these questions diligently. Information will not be copyedited, so please use proper spelling and grammar, use correct capitalization, and avoid abbreviations.

DO NOT FORGET TO SAVE AS PDF _AND_ CLICK THE SUBMIT BUTTON SO YOUR ANSWERS ARE IN OUR DATABASE !!!

Citation Suggestion (if you append the pdf as Appendix we suggest to cite this paper in the caption):
Eysenbach G, CONSORT-EHEALTH Group

CONSORT-EHEALTH: Improving and Standardizing Evaluation Reports of Web-based and Mobile Health Interventions
J Med Internet Res 2011;13(4):e126
URL: http://www.jmir.org/2011/4/e126/
doi: 10.2196/jmir.1923
PMID: 22209829

fernanda.munoz@ufrontera.cl Cambiar de cuenta

Borrador guardado

No compartido

* Indica que la pregunta es obligatoria

Your name *

First Last

Muñoz-Sepúlveda Fernanda

Primary Affiliation (short), City, Country *

University of Toronto, Toronto, Canada

Universidad de La Frontera, Temuco, Chile

Your e-mail address *

abc@gmail.com

fernanda.munoz@ufrontera.cl

Title of your manuscript *

Provide the (draft) title of your manuscript.

WhatsApp-Supported Teledentistry to Reinforce Oral Health Promotion Among Older Adults Residing in Rural and Urban Areas: A Randomized Controlled Trial

Name of your App/Software/Intervention *

If there is a short and a long/alternate name, write the short name first and add the long name in brackets.

Telehealth education intervention (i.e WhatsAp

Evaluated Version (if any)

e.g. "V1", "Release 2017-03-01", "Version 2.0.27913"

Tu respuesta

Language(s) *

What language is the intervention/app in? If multiple languages are available, separate by comma (e.g. "English, French")

Spanish

URL of your Intervention Website or App

e.g. a direct link to the mobile app on app in appstore (itunes, Google Play), or URL of the website. If the intervention is a DVD or hardware, you can also link to an Amazon page.

Tu respuesta

URL of an image/screenshot (optional)

Tu respuesta

Accessibility *

Can an enduser access the intervention presently?

- ( ) access is free and open
- (•) access only for special usergroups, not open
- ( ) access is open to everyone, but requires payment/subscription/in-app purchases
- ( ) app/intervention no longer accessible
- ( ) Otro:

Primary Medical Indication/Disease/Condition *

e.g. "Stress", "Diabetes", or define the target group in brackets after the condition, e.g. "Autism (Parents of children with)", "Alzheimers (Informal Caregivers of)"

Oral health promotion (Community-dwelling ol

Primary Outcomes measured in trial *

comma-separated list of primary outcomes reported in the trial

Oral health knowledge, Attitudes toward oral h

Secondary/other outcomes

Are there any other outcomes the intervention is expected to affect?

Self-reported oral hygiene behaviors, Acceptability of telehealth intervention

Recommended "Dose" *

What do the instructions for users say on how often the app should be used?

- ◯ Approximately Daily
- ◯ Approximately Weekly
- ◯ Approximately Monthly
- ◯ Approximately Yearly
- ◯ "as needed"
- ◉ Otro: Participants were instructed to watch the videos and informed that the

Approx. Percentage of Users (starters) still using the app as recommended after 3 months *

- ◉ unknown / not evaluated
- ◯ 0-10%
- ◯ 11-20%
- ◯ 21-30%
- ◯ 31-40%
- ◯ 41-50%
- ◯ 51-60%
- ◯ 61-70%
- ◯ 71%-80%
- ◯ 81-90%
- ◯ 91-100%
- ◯ Otro:

Overall, was the app/intervention effective? *

- ( ) yes: all primary outcomes were significantly better in intervention group vs control
- (x) partly: SOME primary outcomes were significantly better in intervention group vs control
- ( ) no statistically significant difference between control and intervention
- ( ) potentially harmful: control was significantly better than intervention in one or more outcomes
- ( ) inconclusive: more research is needed
- ( ) Otro:

Article Preparation Status/Stage *

At which stage in your article preparation are you currently (at the time you fill in this form)

- ( ) not submitted yet - in early draft status
- (x) not submitted yet - in late draft status, just before submission
- ( ) submitted to a journal but not reviewed yet
- ( ) submitted to a journal and after receiving initial reviewer comments
- ( ) submitted to a journal and accepted, but not published yet
- ( ) published
- ( ) Otro:

Journal *

If you already know where you will submit this paper (or if it is already submitted), please provide the journal name (if it is not JMIR, provide the journal name under "other")

- ○ not submitted yet / unclear where I will submit this
- ○ Journal of Medical Internet Research (JMIR)
- ◉ JMIR mHealth and UHealth
- ○ JMIR Serious Games
- ○ JMIR Mental Health
- ○ JMIR Public Health
- ○ JMIR Formative Research
- ○ Other JMIR sister journal
- ○ Otro:

Is this a full powered effectiveness trial or a pilot/feasibility trial? *

- ○ Pilot/feasibility
- ◉ Fully powered

Manuscript tracking number *

If this is a JMIR submission, please provide the manuscript tracking number under "other" (The ms tracking number can be found in the submission acknowledgement email, or when you login as author in JMIR. If the paper is already published in JMIR, then the ms tracking number is the four-digit number at the end of the DOI, to be found at the bottom of each published article in JMIR)

- ◉ no ms number (yet) / not (yet) submitted to / published in JMIR
- ○ Otro:

TITLE AND ABSTRACT

1a) TITLE: Identification as a randomized trial in the title

1a) Does your paper address CONSORT item 1a? *

I.e does the title contain the phrase "Randomized Controlled Trial"? (if not, explain the reason under "other")

- (selected) yes
- Otro:

1a-i) Identify the mode of delivery in the title

Identify the mode of delivery. Preferably use “web-based” and/or “mobile” and/or “electronic game” in the title. Avoid ambiguous terms like “online”, “virtual”, “interactive”. Use “Internet-based” only if Intervention includes non-web-based Internet components (e.g. email), use “computer-based” or “electronic” only if offline products are used. Use “virtual” only in the context of “virtual reality” (3-D worlds). Use “online” only in the context of “online support groups”. Complement or substitute product names with broader terms for the class of products (such as “mobile” or “smart phone” instead of “iphone”), especially if the application runs on different platforms.

	1	2	3	4	5	
subitem not at all important	○	○	○	●	○	essential

Borrar selección

Does your paper address subitem 1a-i? *

Copy and paste relevant sections from manuscript title (include quotes in quotation marks "like this" to indicate direct quotes from your manuscript), or elaborate on this item by providing additional information not in the ms, or briefly explain why the item is not applicable/relevant for your study

WhatsApp-Supported Teledentistry to Reinforce Oral Health Promotion Among Older Adults Residing in Rural and Urban Areas: A Randomized Controlled Trial

1a-ii) Non-web-based components or important co-interventions in title

Mention non-web-based components or important co-interventions in title, if any (e.g., “with telephone support”).

	1	2	3	4	5	
subitem not at all important	○	●	○	○	○	essential

Borrar selección

Does your paper address subitem 1a-ii?

Copy and paste relevant sections from manuscript title (include quotes in quotation marks "like this" to indicate direct quotes from your manuscript), or elaborate on this item by providing additional information not in the ms, or briefly explain why the item is not applicable/relevant for your study

WhatsApp-Supported Teledentistry to Reinforce Oral Health Promotion Among Older Adults Residing in Rural and Urban Areas: A Randomized Controlled Trial

1a-iii) Primary condition or target group in the title

Mention primary condition or target group in the title, if any (e.g., “for children with Type I Diabetes”) Example: A Web-based and Mobile Intervention with Telephone Support for Children with Type I Diabetes: Randomized Controlled Trial

	1	2	3	4	5	
subitem not at all important	○	○	○	○	●	essential

Borrar selección

Does your paper address subitem 1a-iii? *

Copy and paste relevant sections from manuscript title (include quotes in quotation marks "like this" to indicate direct quotes from your manuscript), or elaborate on this item by providing additional information not in the ms, or briefly explain why the item is not applicable/relevant for your study

WhatsApp-Supported Teledentistry to Reinforce Oral Health Promotion Among Older Adults Residing in Rural and Urban Areas: A Randomized Controlled Trial

1b) ABSTRACT: Structured summary of trial design, methods, results, and conclusions

NPT extension: Description of experimental treatment, comparator, care providers, centers, and blinding status.

1b-i) Key features/functionalities/components of the intervention and comparator in the METHODS section of the ABSTRACT

Mention key features/functionalities/components of the intervention and comparator in the abstract. If possible, also mention theories and principles used for designing the site. Keep in mind the needs of systematic reviewers and indexers by including important synonyms. (Note: Only report in the abstract what the main paper is reporting. If this information is missing from the main body of text, consider adding it)

	1	2	3	4	5	
subitem not at all important	○	○	○	◉	○	essential

Borrar selección

Does your paper address subitem 1b-i? *

Copy and paste relevant sections from the manuscript abstract (include quotes in quotation marks "like this" to indicate direct quotes from your manuscript), or elaborate on this item by providing additional information not in the ms, or briefly explain why the item is not applicable/relevant for your study

"A baseline clinical evaluation was performed using a teledentistry platform (TEGO®). Participants were randomly assigned in a 1:1 ratio via a computer-based system to either the comparator group, which received a face-to-face oral care instruction session delivered by a dentist, or the test group, which received the same face-to-face education supplemented with telehealth dentistry reinforcement (via WhatsApp and educational videos). Grounded in Social Cognitive Theory (SCT), the telehealth intervention included four validated educational videos covering dental caries, periodontal disease, oral cancer, and dental hygiene"

1b-ii) Level of human involvement in the METHODS section of the ABSTRACT

Clarify the level of human involvement in the abstract, e.g., use phrases like “fully automated” vs. “therapist/nurse/care provider/physician-assisted” (mention number and expertise of providers involved, if any). (Note: Only report in the abstract what the main paper is reporting. If this information is missing from the main body of text, consider adding it)

	1	2	3	4	5	
subitem not at all important	○	○	○	◉	○	essential

Borrar selección

Does your paper address subitem 1b-ii?

Copy and paste relevant sections from the manuscript abstract (include quotes in quotation marks "like this" to indicate direct quotes from your manuscript), or elaborate on this item by providing additional information not in the ms, or briefly explain why the item is not applicable/relevant for your study

"the comparator group, which received a face-to-face oral care instruction session delivered by a dentist, or the test group, which received the same face-to-face education supplemented with telehealth dentistry reinforcement (via WhatsApp and educational videos)"

1b-iii) Open vs. closed, web-based (self-assessment) vs. face-to-face assessments in the METHODS section of the ABSTRACT

Mention how participants were recruited (online vs. offline), e.g., from an open access website or from a clinic or a closed online user group (closed usergroup trial), and clarify if this was a purely web-based trial, or there were face-to-face components (as part of the intervention or for assessment). Clearly say if outcomes were self-assessed through questionnaires (as common in web-based trials). Note: In traditional offline trials, an open trial (open-label trial) is a type of clinical trial in which both the researchers and participants know which treatment is being administered. To avoid confusion, use “blinded” or “unblinded” to indicated the level of blinding instead of “open”, as “open” in web-based trials usually refers to “open access” (i.e. participants can self-enrol). (Note: Only report in the abstract what the main paper is reporting. If this information is missing from the main body of text, consider adding it)

	1	2	3	4	5	
subitem not at all important	○	○	◉	○	○	essential

Borrar selección

Does your paper address subitem 1b-iii?

Copy and paste relevant sections from the manuscript abstract (include quotes in quotation marks "like this" to indicate direct quotes from your manuscript), or elaborate on this item by providing additional information not in the ms, or briefly explain why the item is not applicable/relevant for your study

"A randomized controlled trial was conducted across four municipalities, comprising two urban and two rural areas, with 120 older adults aged 60 and above recruited from regional databases", "Clinicians and statistical advisors were blinded to group allocation, though participants were aware of the intervention during the reinforcement phase."

1b-iv) RESULTS section in abstract must contain use data

Report number of participants enrolled/assessed in each group, the use/uptake of the intervention (e.g., attrition/adherence metrics, use over time, number of logins etc.), in addition to primary/secondary outcomes. (Note: Only report in the abstract what the main paper is reporting. If this information is missing from the main body of text, consider adding it)

	1	2	3	4	5	
subitem not at all important	○	○	○	○	◉	essential

Borrar selección

Does your paper address subitem 1b-iv?

Copy and paste relevant sections from the manuscript abstract (include quotes in quotation marks "like this" to indicate direct quotes from your manuscript), or elaborate on this item by providing additional information not in the ms, or briefly explain why the item is not applicable/relevant for your study

"A total of 103 older adults were analyzed, with 51 in the comparator group and 52 in the test group. Both groups showed significant improvements in knowledge, self-efficacy, and brushing behaviors. The telehealth education group showed significant pre-posttest improvements (P<.05). Treatment differences between the telehealth education and comparator groups were significant for knowledge about dental caries (r= 0.207, IC 95% [0.108, 0.305], P= .036) and attitudes toward oral health (r= 0.281, IC 95% [0.182, 0.379], P=.004). The rural population exhibited greater improvements in recognizing signs and preventive measures for periodontal disease (P=.018) and oral cancer (P<.001), as well as in flossing behavior (P<.001). Overall, the telehealth intervention showed considerable acceptability in this older population (over 80%)."

1b-v) CONCLUSIONS/DISCUSSION in abstract for negative trials

Conclusions/Discussions in abstract for negative trials: Discuss the primary outcome - if the trial is negative (primary outcome not changed), and the intervention was not used, discuss whether negative results are attributable to lack of uptake and discuss reasons. (Note: Only report in the abstract what the main paper is reporting. If this information is missing from the main body of text, consider adding it)

	1	2	3	4	5	
subitem not at all important	○	○	○	○	◉	essential

Borrar selección

Does your paper address subitem 1b-v?

Copy and paste relevant sections from the manuscript abstract (include quotes in quotation marks "like this" to indicate direct quotes from your manuscript), or elaborate on this item by providing additional information not in the ms, or briefly explain why the item is not applicable/relevant for your study

"Both groups showed improvements in oral health indicators, with telehealth reinforcement grounded in SCT, proving effectiveness, especially among the rural population. Telehealth demonstrated acceptability and a strong potential for scaling to diverse contexts and populations."

INTRODUCTION

2a) In INTRODUCTION: Scientific background and explanation of rationale

2a-i) Problem and the type of system/solution

Describe the problem and the type of system/solution that is object of the study: intended as stand-alone intervention vs. incorporated in broader health care program? Intended for a particular patient population? Goals of the intervention, e.g., being more cost-effective to other interventions, replace or complement other solutions? (Note: Details about the intervention are provided in “Methods” under 5)

	1	2	3	4	5	
subitem not at all important	○	○	○	○	◉	essential

Borrar selección

Does your paper address subitem 2a-i? *

Copy and paste relevant sections from the manuscript (include quotes in quotation marks "like this" to indicate direct quotes from your manuscript), or elaborate on this item by providing additional information not in the ms, or briefly explain why the item is not applicable/relevant for your study

"In dentistry, social media-based interventions have effectively reduced gingival index scores and improved oral health knowledge on topics such as tobacco use, oral cancer prevention, and oral hygiene, particularly in younger populations [48]. Expanding these solutions to older adults could significantly enhance their well-being and extend benefits to their families and communities [61]. m-Oral health holds great potential to integrate tailored oral health promotion into the daily routines of older adults through widely used digital platforms, thereby improving their oral health and overall well-being.
Considering all the above, this study aims to develop and evaluate the impact of a theory-driven telehealth education reinforcement intervention targeting rural and urban community-dwelling older adults in the La Araucanía Region, Chile. The intervention leverages m-oral health and social media to improve knowledge, attitudes, and oral self-efficacy towards oral health."

2a-ii) Scientific background, rationale: What is known about the (type of) system

Scientific background, rationale: What is known about the (type of) system that is the object of the study (be sure to discuss the use of similar systems for other conditions/diagnoses, if appropiate), motivation for the study, i.e. what are the reasons for and what is the context for this specific study, from which stakeholder viewpoint is the study performed, potential impact of findings [2]. Briefly justify the choice of the comparator.

	1	2	3	4	5	
subitem not at all important	○	○	○	○	◉	essential

Borrar selección

Does your paper address subitem 2a-ii? *

Copy and paste relevant sections from the manuscript (include quotes in quotation marks "like this" to indicate direct quotes from your manuscript), or elaborate on this item by providing additional information not in the ms, or briefly explain why the item is not applicable/relevant for your study

"Social media, especially when integrated with telehealth education, allows patients to access instructions as needed, enhancing their comfort, independence and potentially contributing to improved well-being [54,56]. Among social media platforms, WhatsApp has been extensively employed in healthcare settings as part of health education interventions. These interventions have demonstrated positive outcomes, highlighting the platform's potential for managing chronic conditions and advancing health education [57]. Examples of reinforcement interventions delivered via WhatsApp include promoting medication adherence [58], supporting Type 2 diabetes management [59], and increasing the uptake of Papanicolaou smear [60]. "

2b) In INTRODUCTION: Specific objectives or hypotheses

Does your paper address CONSORT subitem 2b? *

Copy and paste relevant sections from the manuscript (include quotes in quotation marks "like this" to indicate direct quotes from your manuscript), or elaborate on this item by providing additional information not in the ms, or briefly explain why the item is not applicable/relevant for your study

"this study aims to develop and evaluate the impact of a theory-driven telehealth education reinforcement intervention targeting rural and urban community-dwelling older adults in the La Araucanía Region, Chile. The intervention leverages m-oral health and social media to improve knowledge, attitudes, and oral self-efficacy towards oral health. "

METHODS

3a) Description of trial design (such as parallel, factorial) including allocation ratio

Does your paper address CONSORT subitem 3a? *

Copy and paste relevant sections from the manuscript (include quotes in quotation marks "like this" to indicate direct quotes from your manuscript), or elaborate on this item by providing additional information not in the ms, or briefly explain why the item is not applicable/relevant for your study

"This study was a single center, randomized, controlled clinical trial with parallel design to evaluate the impact of two health education interventions: one using conventional face-to-face, and another using ICT as a reinforcement method". "Participants were randomly allocated to either the Comparator or the Telehealth Education group in a 1:1 ratio using a randomization sequence through the EPIDAT random number generator [76], stratified by each age group."

3b) Important changes to methods after trial commencement (such as eligibility criteria), with reasons

Does your paper address CONSORT subitem 3b? *

Copy and paste relevant sections from the manuscript (include quotes in quotation marks "like this" to indicate direct quotes from your manuscript), or elaborate on this item by providing additional information not in the ms, or briefly explain why the item is not applicable/relevant for your study

It doesn't apply; no modifications were made to the methodology

3b-i) Bug fixes, Downtimes, Content Changes

Bug fixes, Downtimes, Content Changes: ehealth systems are often dynamic systems. A description of changes to methods therefore also includes important changes made on the intervention or comparator during the trial (e.g., major bug fixes or changes in the functionality or content) (5-iii) and other “unexpected events” that may have influenced study design such as staff changes, system failures/downtimes, etc. [2].

	1	2	3	4	5	
subitem not at all important	○	○	○	○	◉	essential

Borrar selección

Does your paper address subitem 3b-i?

Copy and paste relevant sections from the manuscript (include quotes in quotation marks "like this" to indicate direct quotes from your manuscript), or elaborate on this item by providing additional information not in the ms, or briefly explain why the item is not applicable/relevant for your study

It doesn't apply; no modifications were made to the methodology

4a) Eligibility criteria for participants

Does your paper address CONSORT subitem 4a? *

Copy and paste relevant sections from the manuscript (include quotes in quotation marks "like this" to indicate direct quotes from your manuscript), or elaborate on this item by providing additional information not in the ms, or briefly explain why the item is not applicable/relevant for your study

"The study included participants of all genders, aged 60 years and older, who were functionally independent (Barthel index ≥ 90), capable of following instructions, and had access to a smartphone with internet connectivity. Key exclusion criteria included cognitive impairment (Mini-Mental state examination <25), edentulism, and an inability to use WhatsApp."

4a-i) Computer / Internet literacy

Computer / Internet literacy is often an implicit “de facto” eligibility criterion - this should be explicitly clarified.

	1	2	3	4	5	
subitem not at all important	○	○	○	○	◉	essential

Borrar selección

Does your paper address subitem 4a-i?

Copy and paste relevant sections from the manuscript (include quotes in quotation marks "like this" to indicate direct quotes from your manuscript), or elaborate on this item by providing additional information not in the ms, or briefly explain why the item is not applicable/relevant for your study

"Key exclusion criteria included cognitive impairment (Mini-Mental state examination <25), edentulism, and an inability to use WhatsApp."

4a-ii) Open vs. closed, web-based vs. face-to-face assessments:

Open vs. closed, web-based vs. face-to-face assessments: Mention how participants were recruited (online vs. offline), e.g., from an open access website or from a clinic, and clarify if this was a purely web-based trial, or there were face-to-face components (as part of the intervention or for assessment), i.e., to what degree got the study team to know the participant. In online-only trials, clarify if participants were quasi-anonymous and whether having multiple identities was possible or whether technical or logistical measures (e.g., cookies, email confirmation, phone calls) were used to detect/prevent these.

	1	2	3	4	5	
subitem not at all important	○	○	○	○	◉	essential

Borrar selección

Does your paper address subitem 4a-ii? *

Copy and paste relevant sections from the manuscript (include quotes in quotation marks "like this" to indicate direct quotes from your manuscript), or elaborate on this item by providing additional information not in the ms, or briefly explain why the item is not applicable/relevant for your study

"Recruitment of the participants was conducted via phone using databases from the regional branch of the National Senior Citizen Service (SENAMA, Chile) and community organizations, selecting individuals based on specific criteria and their capability to attend appointments at a mobile dental clinic located in the municipalities of Freire, Temuco (the regional capital), Angol, and Los Sauces. " ". An independent researcher, uninvolved in the recruitment or intervention, managed the allocation sequence. Once participants were deemed eligible, provided informed consent, and completed the face-to-face education session, the principal investigator contacted the independent researcher to obtain the corresponding group assignment.
To prevent contamination and cross-over between treatment arms, the general dentist, outcome assessor, and data analysis were blinded to group assignments, though participants were aware of the intervention during the reinforcement phase. All participants also signed an agreement committing not to share the videos with family or friends if they received them. "

4a-iii) Information giving during recruitment

Information given during recruitment. Specify how participants were briefed for recruitment and in the informed consent procedures (e.g., publish the informed consent documentation as appendix, see also item X26), as this information may have an effect on user self-selection, user expectation and may also bias results.

	1	2	3	4	5	
subitem not at all important	○	○	◉	○	○	essential

Borrar selección

Does your paper address subitem 4a-iii?

Copy and paste relevant sections from the manuscript (include quotes in quotation marks "like this" to indicate direct quotes from your manuscript), or elaborate on this item by providing additional information not in the ms, or briefly explain why the item is not applicable/relevant for your study

"Participants received general information about study activities, including educational content delivery, survey completion at three time points, and their right to withdraw at any time. Specific study aims were withheld to avoid bias, per CIOMS Guideline 10[68]. "

4b) Settings and locations where the data were collected

Does your paper address CONSORT subitem 4b? *

Copy and paste relevant sections from the manuscript (include quotes in quotation marks "like this" to indicate direct quotes from your manuscript), or elaborate on this item by providing additional information not in the ms, or briefly explain why the item is not applicable/relevant for your study

"capability to attend appointments at a mobile dental clinic located in the municipalities of Freire, Temuco (the regional capital), Angol, and Los Sauces. "

4b-i) Report if outcomes were (self-)assessed through online questionnaires

Clearly report if outcomes were (self-)assessed through online questionnaires (as common in web-based trials) or otherwise.

	1	2	3	4	5	
subitem not at all important	○	○	○	○	◉	essential

Borrar selección

Does your paper address subitem 4b-i? *

Copy and paste relevant sections from the manuscript (include quotes in quotation marks "like this" to indicate direct quotes from your manuscript), or elaborate on this item by providing additional information not in the ms, or briefly explain why the item is not applicable/relevant for your study

"The study comprised four phases:
1. Enrollment and Pretest: Participants were enrolled and completed a pretest via telephone
2. Clinical evaluation and face-to-face educational intervention in a mobile dental clinic
3. Telehealth Educational intervention: Participants of the Telehealth Education group received educational videos via WhatsApp.
4. Post-test evaluations: Post-test evaluations were administered via telephone six weeks after each group had received their respective interventions."

4b-ii) Report how institutional affiliations are displayed

Report how institutional affiliations are displayed to potential participants [on ehealth media], as affiliations with prestigious hospitals or universities may affect volunteer rates, use, and reactions with regards to an intervention.(Not a required item – describe only if this may bias results)

	1	2	3	4	5	
subitem not at all important	○	○	○	○	○	essential

Does your paper address subitem 4b-ii?

Copy and paste relevant sections from the manuscript (include quotes in quotation marks "like this" to indicate direct quotes from your manuscript), or elaborate on this item by providing additional information not in the ms, or briefly explain why the item is not applicable/relevant for your study

Tu respuesta

5) The interventions for each group with sufficient details to allow replication, including how and when they were actually administered

5-i) Mention names, credential, affiliations of the developers, sponsors, and owners

Mention names, credential, affiliations of the developers, sponsors, and owners [6] (if authors/evaluators are owners or developer of the software, this needs to be declared in a “Conflict of interest” section or mentioned elsewhere in the manuscript).

	1	2	3	4	5	
subitem not at all important	○	○	○	○	◉	essential

Borrar selección

Does your paper address subitem 5-i?

Copy and paste relevant sections from the manuscript (include quotes in quotation marks "like this" to indicate direct quotes from your manuscript), or elaborate on this item by providing additional information not in the ms, or briefly explain why the item is not applicable/relevant for your study

"Comparator group (Face-to-face intervention): The face-to-face educational session was individually conducted by a previously trained general dentist, following a standardized 15-minute presentation. A practical demonstration of dental floss usage and brushing technique was performed on a dental model and in the participant´s oral cavity. This hands-on approach enables participants to acquire the skills necessary for independent oral hygiene practices through observation and experience, with feedback provided to enhance their abilities to execute the action. According to SCT, watching others engage in a behavior influences an observer's self-efficacy, influencing their perceptions of personal ability, expected outcomes, and providing strategies for effective performance [22].

Telehealth education group: In addition to the face-to-face session, this group received four educational videos on oral health promotion delivered via WhatsApp. Following the initial session, participants received two five-minute videos per week via WhatsApp, with links directing them to YouTube for viewing. Participants were instructed to watch the videos and informed that their opinions would be requested later, but no specific guidance was provided on the frequency or manner of viewing"

5-ii) Describe the history/development process

Describe the history/development process of the application and previous formative evaluations (e.g., focus groups, usability testing), as these will have an impact on adoption/use rates and help with interpreting results.

	1	2	3	4	5	
subitem not at all important	○	○	○	○	○	essential

Does your paper address subitem 5-ii?

Copy and paste relevant sections from the manuscript (include quotes in quotation marks "like this" to indicate direct quotes from your manuscript), or elaborate on this item by providing additional information not in the ms, or briefly explain why the item is not applicable/relevant for your study

"A pilot study in a rural community in La Araucanía (n=76) identified oral health knowledge gaps, guiding the development of targeted educational modules on self-care, dental caries, periodontal disease, and oral cancer.
The interventions were designed based on Social Cognitive Theory (SCT), aimed at promoting behavioral change through constructs such as self-efficacy, observational learning, behavioral capability, reinforcement, and outcome expectations [22]. The educational materials adhered to established clinical guidelines [70–73], and focused on oral hygiene practices (brushing and flossing), disease prevention and symptom recognition.
To ensure quality, the materials were validated by an expert panel of eight academics using the Item Content Validity Index (I-CVI) and the Patient Education Materials Assessment Tool for Audiovisual Materials (PEMAT A/V). The I-CVI assessed the relevance of script items on a 4-point scale, with scores 3 or 4 deemed acceptable[74]. An I-CVI of 0.83 or higher was considered appropriate [74]. The PEMAT A/V evaluated the understandability (13 items) and actionability (4 items) of audiovisual materials for patients, with scores expressed as percentages [75]. A PEMAT A/V score of 70% or below indicated inadequate understandability or actionability [75].
Pre-existing educational videos on oral hygiene and oral cancer prevention achieved high levels of understandability for oral hygiene (91%) and oral cancer (87%), as well as actionability for both (100%)[75]. Some aspects, such as text readability (71%), clarity in defining clinical terms (75%), and presentation of informational headings (81%), were improved based on panel feedback.
Scripts for new videos on dental caries and periodontal disease prevention scored 100% in apparent validation and achieved an I-CVI of 1.0, except for the item "risk factors for periodontal disease" (I-CVI 0.88). Final videos were rated highly in understandability for dental caries (97%) and periodontal disease (98%), as well as actionability for both (100%) [75].
The videos featured older adults sharing narratives about how they effectively addressed or improved their oral health concerns, alongside a dental specialist showcasing practical recommendations. A standardized PowerPoint presentation was also developed to align with the video content."

5-iii) Revisions and updating

Revisions and updating. Clearly mention the date and/or version number of the application/intervention (and comparator, if applicable) evaluated, or describe whether the intervention underwent major changes during the evaluation process, or whether the development and/or content was "frozen" during the trial. Describe dynamic components such as news feeds or changing content which may have an impact on the replicability of the intervention (for unexpected events see item 3b).

	1	2	3	4	5	
subitem not at all important	○	○	◉	○	○	essential

Borrar selección

Does your paper address subitem 5-iii?

Copy and paste relevant sections from the manuscript (include quotes in quotation marks "like this" to indicate direct quotes from your manuscript), or elaborate on this item by providing additional information not in the ms, or briefly explain why the item is not applicable/relevant for your study

Is not applicable to this study, the intervention didn´t change

5-iv) Quality assurance methods

Provide information on quality assurance methods to ensure accuracy and quality of information provided [1], if applicable.

	1	2	3	4	5	
subitem not at all important	○	○	○	○	○	essential

Does your paper address subitem 5-iv?

Copy and paste relevant sections from the manuscript (include quotes in quotation marks "like this" to indicate direct quotes from your manuscript), or elaborate on this item by providing additional information not in the ms, or briefly explain why the item is not applicable/relevant for your study

Tu respuesta

5-v) Ensure replicability by publishing the source code, and/or providing screenshots/screen-capture video, and/or providing flowcharts of the algorithms used

Ensure replicability by publishing the source code, and/or providing screenshots/screen-capture video, and/or providing flowcharts of the algorithms used. Replicability (i.e., other researchers should in principle be able to replicate the study) is a hallmark of scientific reporting.

	1	2	3	4	5	
subitem not at all important	○	○	○	●	○	essential

Borrar selección

Does your paper address subitem 5-v?

Copy and paste relevant sections from the manuscript (include quotes in quotation marks "like this" to indicate direct quotes from your manuscript), or elaborate on this item by providing additional information not in the ms, or briefly explain why the item is not applicable/relevant for your study

The phases of educational material development and the theory upon which it was based were provided

5-vi) Digital preservation

Digital preservation: Provide the URL of the application, but as the intervention is likely to change or disappear over the course of the years; also make sure the intervention is archived (Internet Archive, webcitation.org, and/or publishing the source code or screenshots/videos alongside the article). As pages behind login screens cannot be archived, consider creating demo pages which are accessible without login.

	1	2	3	4	5	
subitem not at all important	○	○	◉	○	○	essential

Borrar selección

Does your paper address subitem 5-vi?

Copy and paste relevant sections from the manuscript (include quotes in quotation marks "like this" to indicate direct quotes from your manuscript), or elaborate on this item by providing additional information not in the ms, or briefly explain why the item is not applicable/relevant for your study

Tu respuesta

5-vii) Access

Access: Describe how participants accessed the application, in what setting/context, if they had to pay (or were paid) or not, whether they had to be a member of specific group. If known, describe how participants obtained "access to the platform and Internet" [1]. To ensure access for editors/reviewers/readers, consider to provide a "backdoor" login account or demo mode for reviewers/readers to explore the application (also important for archiving purposes, see vi).

	1	2	3	4	5	
subitem not at all important	○	○	○	○	◉	essential

Borrar selección

Does your paper address subitem 5-vii? *

Copy and paste relevant sections from the manuscript (include quotes in quotation marks "like this" to indicate direct quotes from your manuscript), or elaborate on this item by providing additional information not in the ms, or briefly explain why the item is not applicable/relevant for your study

"Following the initial session, participants received two five-minute videos per week via WhatsApp, with links directing them to YouTube for viewing. Participants were instructed to watch the videos and informed that their opinions would be requested later, but no specific guidance was provided on the frequency or manner of viewing."

5-viii) Mode of delivery, features/functionalities/components of the intervention and comparator, and the theoretical framework

Describe mode of delivery, features/functionalities/components of the intervention and comparator, and the theoretical framework [6] used to design them (instructional strategy [1], behaviour change techniques, persuasive features, etc., see e.g., [7, 8] for terminology). This includes an in-depth description of the content (including where it is coming from and who developed it) [1],” whether [and how] it is tailored to individual circumstances and allows users to track their progress and receive feedback” [6]. This also includes a description of communication delivery channels and – if computer-mediated communication is a component – whether communication was synchronous or asynchronous [6]. It also includes information on presentation strategies [1], including page design principles, average amount of text on pages, presence of hyperlinks to other resources, etc. [1].

	1	2	3	4	5	
subitem not at all important	○	○	○	●	○	essential

Borrar selección

Does your paper address subitem 5-viii? *

Copy and paste relevant sections from the manuscript (include quotes in quotation marks "like this" to indicate direct quotes from your manuscript), or elaborate on this item by providing additional information not in the ms, or briefly explain why the item is not applicable/relevant for your study

"The interventions were designed based on Social Cognitive Theory (SCT), aimed at promoting behavioral change through constructs such as self-efficacy, observational learning, behavioral capability, reinforcement, and outcome expectations [22]. The educational materials adhered to established clinical guidelines [70–73], and focused on oral hygiene practices (brushing and flossing), disease prevention and symptom recognition.

To ensure quality, the materials were validated by an expert panel of eight academics using the Item Content Validity Index (I-CVI) and the Patient Education Materials Assessment Tool for Audiovisual Materials (PEMAT A/V). The I-CVI assessed the relevance of script items on a 4-point scale, with scores 3 or 4 deemed acceptable[74]. An I-CVI of 0.83 or higher was considered appropriate [74]. The PEMAT A/V evaluated the understandability (13 items) and actionability (4 items) of audiovisual materials for patients, with scores expressed as percentages [75]. A PEMAT A/V score of 70% or below indicated inadequate understandability or actionability [75].

Pre-existing educational videos on oral hygiene and oral cancer prevention achieved high levels of understandability for oral hygiene (91%) and oral cancer (87%), as well as actionability for both (100%)[75]. Some aspects, such as text readability (71%), clarity in defining clinical terms (75%), and presentation of informational headings (81%), were improved based on panel feedback.

Scripts for new videos on dental caries and periodontal disease prevention scored 100% in apparent validation and achieved an I-CVI of 1.0, except for the item "risk factors for periodontal disease" (I-CVI 0.88). Final videos were rated highly in understandability for dental caries (97%) and periodontal disease (98%), as well as actionability for both (100%) [75].

The videos featured older adults sharing narratives about how they effectively addressed or improved their oral health concerns, alongside a dental specialist showcasing practical recommendations. A standardized PowerPoint presentation was also developed to align with the video content.

"

5-ix) Describe use parameters

Describe use parameters (e.g., intended “doses” and optimal timing for use). Clarify what instructions or recommendations were given to the user, e.g., regarding timing, frequency, heaviness of use, if any, or was the intervention used ad libitum.

	1	2	3	4	5	
subitem not at all important	○	○	○	○	◉	essential

Borrar selección

Does your paper address subitem 5-ix?

Copy and paste relevant sections from the manuscript (include quotes in quotation marks "like this" to indicate direct quotes from your manuscript), or elaborate on this item by providing additional information not in the ms, or briefly explain why the item is not applicable/relevant for your study

"Participants were instructed to watch the videos and informed that their opinions would be requested later, but no specific guidance was provided on the frequency or manner of viewing."

5-x) Clarify the level of human involvement

Clarify the level of human involvement (care providers or health professionals, also technical assistance) in the e-intervention or as co-intervention (detail number and expertise of professionals involved, if any, as well as “type of assistance offered, the timing and frequency of the support, how it is initiated, and the medium by which the assistance is delivered”. It may be necessary to distinguish between the level of human involvement required for the trial, and the level of human involvement required for a routine application outside of a RCT setting (discuss under item 21 – generalizability).

	1	2	3	4	5	
subitem not at all important	○	○	◉	○	○	essential

Borrar selección

Does your paper address subitem 5-x?

Copy and paste relevant sections from the manuscript (include quotes in quotation marks "like this" to indicate direct quotes from your manuscript), or elaborate on this item by providing additional information not in the ms, or briefly explain why the item is not applicable/relevant for your study

"Comparator group (Face-to-face intervention): The face-to-face educational session was individually conducted by a previously trained general dentist, following a standardized 15-minute presentation. A practical demonstration of dental floss usage and brushing technique was performed on a dental model and in the participant´s oral cavity. This hands-on approach enables participants to acquire the skills necessary for independent oral hygiene practices through observation and experience, with feedback provided to enhance their abilities to execute the action. According to SCT, watching others engage in a behavior influences an observer's self-efficacy, influencing their perceptions of personal ability, expected outcomes, and providing strategies for effective performance [22].

Telehealth education group: In addition to the face-to-face session, this group received four educational videos on oral health promotion delivered via WhatsApp. Following the initial session, participants received two five-minute videos per week via WhatsApp, with links directing them to YouTube for viewing. Participants were instructed to watch the videos and informed that their opinions would be requested later, but no specific guidance was provided on the frequency or manner of viewing.
"

5-xi) Report any prompts/reminders used

Report any prompts/reminders used: Clarify if there were prompts (letters, emails, phone calls, SMS) to use the application, what triggered them, frequency etc. It may be necessary to distinguish between the level of prompts/reminders required for the trial, and the level of prompts/reminders for a routine application outside of a RCT setting (discuss under item 21 – generalizability).

	1	2	3	4	5	
subitem not at all important	○	○	○	○	◉	essential

Borrar selección

Does your paper address subitem 5-xi? *

Copy and paste relevant sections from the manuscript (include quotes in quotation marks "like this" to indicate direct quotes from your manuscript), or elaborate on this item by providing additional information not in the ms, or briefly explain why the item is not applicable/relevant for your study

No reminders were provided for watching the videos

5-xii) Describe any co-interventions (incl. training/support)

Describe any co-interventions (incl. training/support): Clearly state any interventions that are provided in addition to the targeted eHealth intervention, as ehealth intervention may not be designed as stand-alone intervention. This includes training sessions and support [1]. It may be necessary to distinguish between the level of training required for the trial, and the level of training for a routine application outside of a RCT setting (discuss under item 21 – generalizability.

	1	2	3	4	5	
subitem not at all important	○	○	○	●	○	essential

Borrar selección

Does your paper address subitem 5-xii? *

Copy and paste relevant sections from the manuscript (include quotes in quotation marks "like this" to indicate direct quotes from your manuscript), or elaborate on this item by providing additional information not in the ms, or briefly explain why the item is not applicable/relevant for your study

"The face-to-face educational session was individually conducted by a previously trained general dentist, following a standardized 15-minute presentation. A practical demonstration of dental floss usage and brushing technique was performed on a dental model and in the participant´s oral cavity. This hands-on approach enables participants to acquire the skills necessary for independent oral hygiene practices through observation and experience, with feedback provided to enhance their abilities to execute the action. According to SCT, watching others engage in a behavior influences an observer's self-efficacy, influencing their perceptions of personal ability, expected outcomes, and providing strategies for effective performance [22]".

6a) Completely defined pre-specified primary and secondary outcome measures, including how and when they were assessed

Does your paper address CONSORT subitem 6a? *

Copy and paste relevant sections from the manuscript (include quotes in quotation marks "like this" to indicate direct quotes from your manuscript), or elaborate on this item by providing additional information not in the ms, or briefly explain why the item is not applicable/relevant for your study

Educational interventions were assessed using pre- and six-week post-intervention questionnaires, which evaluated each component of the oral health knowledge (i.e., dental caries, periodontal disease, oral cancer and the cumulative score), attitudes, and oral self-efficacy questionnaire. Additionally, these results were compared between both groups and divided by their place of residency. Secondary outcomes included self-reported oral hygiene behaviors and the perceived acceptability of the telehealth education strategy.

6a-i) Online questionnaires: describe if they were validated for online use and apply CHERRIES items to describe how the questionnaires were designed/deployed

If outcomes were obtained through online questionnaires, describe if they were validated for online use and apply CHERRIES items to describe how the questionnaires were designed/deployed [9].

	1	2	3	4	5	
subitem not at all important	○	○	○	○	○	essential

Does your paper address subitem 6a-i?

Copy and paste relevant sections from manuscript text

Tu respuesta

6a-ii) Describe whether and how “use” (including intensity of use/dosage) was defined/measured/monitored

Describe whether and how “use” (including intensity of use/dosage) was defined/measured/monitored (logins, logfile analysis, etc.). Use/adoption metrics are important process outcomes that should be reported in any ehealth trial.

	1	2	3	4	5	
subitem not at all important	○	○	◉	○	○	essential

Borrar selección

Does your paper address subitem 6a-ii?

Copy and paste relevant sections from manuscript text

" Participants were instructed to watch the videos and informed that their opinions would be requested later, but no specific guidance was provided on the frequency or manner of viewing."

6a-iii) Describe whether, how, and when qualitative feedback from participants was obtained

Describe whether, how, and when qualitative feedback from participants was obtained (e.g., through emails, feedback forms, interviews, focus groups).

	1	2	3	4	5	
subitem not at all important	○	○	◉	○	○	essential

Borrar selección

Does your paper address subitem 6a-iii?

Copy and paste relevant sections from manuscript text

6 weeks post intervention through telephone applied surveys

6b) Any changes to trial outcomes after the trial commenced, with reasons

Does your paper address CONSORT subitem 6b? *

Copy and paste relevant sections from the manuscript (include quotes in quotation marks "like this" to indicate direct quotes from your manuscript), or elaborate on this item by providing additional information not in the ms, or briefly explain why the item is not applicable/relevant for your study

Not applicable, as there were no changes in the outcomes

7a) How sample size was determined

NPT: When applicable, details of whether and how the clustering by care provides or centers was addressed

7a-i) Describe whether and how expected attrition was taken into account when calculating the sample size

Describe whether and how expected attrition was taken into account when calculating the sample size.

	1	2	3	4	5	
subitem not at all important	○	○	○	○	◉	essential

Borrar selección

Does your paper address subitem 7a-i?

Copy and paste relevant sections from manuscript title (include quotes in quotation marks "like this" to indicate direct quotes from your manuscript), or elaborate on this item by providing additional information not in the ms, or briefly explain why the item is not applicable/relevant for your study

"The sample size was determined using the G*Power 3.1.9.7 software for Windows to test the differences between two independent means. With a significance of 0.05, an effect size of 0.5 and a power of 0.8, the minimum sample size was calculated to be 102. Considering potential withdrawals, the study included 120 participants, with 60 in each group [69]. Stratified random sampling was conducted, considering age categorization (60-69 years, 70-79 years, and 80 years and over) and geographic sector, adjusted based on the percentage of older adults with smartphone access [53]"

7b) When applicable, explanation of any interim analyses and stopping guidelines

Does your paper address CONSORT subitem 7b? *

Copy and paste relevant sections from the manuscript (include quotes in quotation marks "like this" to indicate direct quotes from your manuscript), or elaborate on this item by providing additional information not in the ms, or briefly explain why the item is not applicable/relevant for your study

Not applicable, as there were no interim analyses

8a) Method used to generate the random allocation sequence

NPT: When applicable, how care providers were allocated to each trial group

Does your paper address CONSORT subitem 8a? *

Copy and paste relevant sections from the manuscript (include quotes in quotation marks "like this" to indicate direct quotes from your manuscript), or elaborate on this item by providing additional information not in the ms, or briefly explain why the item is not applicable/relevant for your study

"Participants were randomly allocated to either the Comparator or the Telehealth Education group in a 1:1 ratio using a randomization sequence through the EPIDAT random number generator [76], stratified by each age group"

8b) Type of randomisation; details of any restriction (such as blocking and block size)

Does your paper address CONSORT subitem 8b? *

Copy and paste relevant sections from the manuscript (include quotes in quotation marks "like this" to indicate direct quotes from your manuscript), or elaborate on this item by providing additional information not in the ms, or briefly explain why the item is not applicable/relevant for your study

"Participants were randomly allocated to either the Comparator or the Telehealth Education group in a 1:1 ratio using a randomization sequence through the EPIDAT random number generator [76], stratified by each age group"

9) Mechanism used to implement the random allocation sequence (such as sequentially numbered containers), describing any steps taken to conceal the sequence until interventions were assigned

Does your paper address CONSORT subitem 9? *

Copy and paste relevant sections from the manuscript (include quotes in quotation marks "like this" to indicate direct quotes from your manuscript), or elaborate on this item by providing additional information not in the ms, or briefly explain why the item is not applicable/relevant for your study

"Participants were randomly allocated to either the Comparator or the Telehealth Education group in a 1:1 ratio using a randomization sequence through the EPIDAT random number generator [76], stratified by each age group. An independent researcher, uninvolved in the recruitment or intervention, managed the allocation sequence. Once participants were deemed eligible, provided informed consent, and completed the face-to-face education session, the principal investigator contacted the independent researcher to obtain the corresponding group assignment.
To prevent contamination and cross-over between treatment arms, the general dentist, outcome assessor, and data analysis were blinded to group assignments, though participants were aware of the intervention during the reinforcement phase. All participants also signed an agreement committing not to share the videos with family or friends if they received them.
"

10) Who generated the random allocation sequence, who enrolled participants, and who assigned participants to interventions

Does your paper address CONSORT subitem 10? *

Copy and paste relevant sections from the manuscript (include quotes in quotation marks "like this" to indicate direct quotes from your manuscript), or elaborate on this item by providing additional information not in the ms, or briefly explain why the item is not applicable/relevant for your study

"Participants were randomly allocated to either the Comparator or the Telehealth Education group in a 1:1 ratio using a randomization sequence through the EPIDAT random number generator [76], stratified by each age group. An independent researcher, uninvolved in the recruitment or intervention, managed the allocation sequence. Once participants were deemed eligible, provided informed consent, and completed the face-to-face education session, the principal investigator contacted the independent researcher to obtain the corresponding group assignment.
To prevent contamination and cross-over between treatment arms, the general dentist, outcome assessor, and data analysis were blinded to group assignments, though participants were aware of the intervention during the reinforcement phase. All participants also signed an agreement committing not to share the videos with family or friends if they received them."

11a) If done, who was blinded after assignment to interventions (for example, participants, care providers, those assessing outcomes) and how

NPT: Whether or not administering co-interventions were blinded to group assignment

11a-i) Specify who was blinded, and who wasn't

Specify who was blinded, and who wasn't. Usually, in web-based trials it is not possible to blind the participants [1, 3] (this should be clearly acknowledged), but it may be possible to blind outcome assessors, those doing data analysis or those administering co-interventions (if any).

	1	2	3	4	5	
subitem not at all important	○	○	○	○	◉	essential

Borrar selección

Does your paper address subitem 11a-i? *

Copy and paste relevant sections from the manuscript (include quotes in quotation marks "like this" to indicate direct quotes from your manuscript), or elaborate on this item by providing additional information not in the ms, or briefly explain why the item is not applicable/relevant for your study

"To prevent contamination and cross-over between treatment arms, the general dentist, outcome assessor, and data analysis were blinded to group assignments, though participants were aware of the intervention during the reinforcement phase. All participants also signed an agreement committing not to share the videos with family or friends if they received them. "

11a-ii) Discuss e.g., whether participants knew which intervention was the “intervention of interest” and which one was the “comparator”

Informed consent procedures (4a-ii) can create biases and certain expectations - discuss e.g., whether participants knew which intervention was the “intervention of interest” and which one was the “comparator”.

	1	2	3	4	5	
subitem not at all important	○	○	○	○	◉	essential

Borrar selección

Does your paper address subitem 11a-ii?

Copy and paste relevant sections from the manuscript (include quotes in quotation marks "like this" to indicate direct quotes from your manuscript), or elaborate on this item by providing additional information not in the ms, or briefly explain why the item is not applicable/relevant for your study

"Participants received general information about study activities, including educational content delivery, survey completion at three time points, and their right to withdraw at any time. Specific study aims were withheld to avoid bias, per CIOMS Guideline 10[68]. All participants also signed an agreement committing not to share the videos with family or friends if they received them. "

11b) If relevant, description of the similarity of interventions
(this item is usually not relevant for ehealth trials as it refers to similarity of a placebo or sham intervention to a active medication/intervention)

Does your paper address CONSORT subitem 11b? *

Copy and paste relevant sections from the manuscript (include quotes in quotation marks "like this" to indicate direct quotes from your manuscript), or elaborate on this item by providing additional information not in the ms, or briefly explain why the item is not applicable/relevant for your study

"Comparator group (Face-to-face intervention): The face-to-face educational session was individually conducted by a previously trained general dentist, following a standardized 15-minute presentation. A practical demonstration of dental floss usage and brushing technique was performed on a dental model and in the participant´s oral cavity. This hands-on approach enables participants to acquire the skills necessary for independent oral hygiene practices through observation and experience, with feedback provided to enhance their abilities to execute the action. According to SCT, watching others engage in a behavior influences an observer's self-efficacy, influencing their perceptions of personal ability, expected outcomes, and providing strategies for effective performance [22].

Telehealth education group: In addition to the face-to-face session, this group received four educational videos on oral health promotion delivered via WhatsApp. Following the initial session, participants received two five-minute videos per week via WhatsApp, with links directing them to YouTube for viewing. Participants were instructed to watch the videos and informed that their opinions would be requested later, but no specific guidance was provided on the frequency or manner of viewing.
"

12a) Statistical methods used to compare groups for primary and secondary outcomes
NPT: When applicable, details of whether and how the clustering by care providers or centers was addressed

Does your paper address CONSORT subitem 12a? *

Copy and paste relevant sections from the manuscript (include quotes in quotation marks "like this" to indicate direct quotes from your manuscript), or elaborate on this item by providing additional information not in the ms, or briefly explain why the item is not applicable/relevant for your study

"Only participants who completed the study were included in the statistical analysis, following a per-protocol approach focused on adherence to study procedures. Descriptive analyses were conducted for sociodemographic variables (age, sex, RSH, place of residency, educational level, and smartphone usage), clinical data (frailty, depression, oral health habits, number of remaining teeth), and components of the oral health knowledge, attitudes, and self-efficacy questionnaire, were presented as percentages, means and standard deviations.

The oral health knowledge, attitudes, and self-efficacy questionnaire was disaggregated into its individual components to describe the results for each. Scores were tested for normality using Anderson-Darling and D'Agostino tests. Group-level changes were analyzed using paired t-tests or Wilcoxon signed-rank tests, depending on data normality. Additionally, post-test scores between the two intervention groups were compared using independent t-tests or Mann-Whitney U tests, as appropriate. Key results were reported in terms of effect sizes, 95% confidence intervals, and statistical significance.

For each question in the questionnaire, the percentage of correct responses by disease (dental caries, periodontal disease, and oral cancer) was calculated, stratified by intervention group and place of residence (urban or rural).

The acceptability of telehealth education was assessed using a Likert scale to measure the perceived usefulness of the educational video, a dichotomous question regarding the recommendation of the educational strategy, and the study dropout rate. Acceptability results were presented as percentages.

Additionally, Multiple Correspondence Analysis (MCA) was employed to explore associations between post-pre-intervention score differences in the telehealth education group and sociodemographic and clinical factors collected on the TEGO® platform. This analysis identified patterns and relationships among sociodemographic variables (e.g., age, educational level, place of residence, and smartphone use), clinical variables (e.g., functional dentition, defined as ≥20 teeth, versus non-functional dentition, defined as <20 teeth), and score differences between pre- and post-intervention. MCA is an exploratory statistical technique that facilitates the visualization of complex relationships among categorical variables.

Score differences were categorized into three subgroups based on findings from previous studies [33,34,81]: significant improvement (≥50% improvement in component scores), moderate improvement (20-49% improvement), and no change or deterioration. This approach provided a deeper understanding of how sociodemographic and clinical factors might have influenced intervention outcomes.

Data analysis was conducted using IBM SPSS Statistics software (version 23.0) and complemented with GraphPad Prism (version 8) for graphical representation. A p-value <.05 was set as the threshold for statistical significance.

"

12a-i) Imputation techniques to deal with attrition / missing values

Imputation techniques to deal with attrition / missing values: Not all participants will use the intervention/comparator as intended and attrition is typically high in ehealth trials. Specify how participants who did not use the application or dropped out from the trial were treated in the statistical analysis (a complete case analysis is strongly discouraged, and simple imputation techniques such as LOCF may also be problematic [4]).

	1	2	3	4	5	
subitem not at all important	○	○	○	○	◉	essential

Borrar selección

Does your paper address subitem 12a-i? *

Copy and paste relevant sections from the manuscript (include quotes in quotation marks "like this" to indicate direct quotes from your manuscript), or elaborate on this item by providing additional information not in the ms, or briefly explain why the item is not applicable/relevant for your study

"Only participants who completed the study were included in the statistical analysis, following a per-protocol approach focused on adherence to study procedures. "

12b) Methods for additional analyses, such as subgroup analyses and adjusted analyses

Does your paper address CONSORT subitem 12b? *

Copy and paste relevant sections from the manuscript (include quotes in quotation marks "like this" to indicate direct quotes from your manuscript), or elaborate on this item by providing additional information not in the ms, or briefly explain why the item is not applicable/relevant for your study

"For each question in the questionnaire, the percentage of correct responses by disease (dental caries, periodontal disease, and oral cancer) was calculated, stratified by intervention group and place of residence (urban or rural)."

X26) REB/IRB Approval and Ethical Considerations [recommended as subheading under "Methods"] (not a CONSORT item)

X26-i) Comment on ethics committee approval

	1	2	3	4	5	
subitem not at all important	○	○	○	○	◉	essential

Borrar selección

Does your paper address subitem X26-i?

Copy and paste relevant sections from the manuscript (include quotes in quotation marks "like this" to indicate direct quotes from your manuscript), or elaborate on this item by providing additional information not in the ms, or briefly explain why the item is not applicable/relevant for your study

"The trial was approved by the Research Ethics Committee of the Universidad de La Frontera, Temuco, Chile (Folio Number 082/22), and was conducted in accordance with the Declaration of Helsinki. All participants provided written informed consent. The trial has been registered at ClinicalTrials.gov [NCT05917548]."

x26-ii) Outline informed consent procedures

Outline informed consent procedures e.g., if consent was obtained offline or online (how? Checkbox, etc.?), and what information was provided (see 4a-ii). See [6] for some items to be included in informed consent documents.

	1	2	3	4	5	
subitem not at all important	○	○	○	○	◉	essential

Borrar selección

Does your paper address subitem X26-ii?

Copy and paste relevant sections from the manuscript (include quotes in quotation marks "like this" to indicate direct quotes from your manuscript), or elaborate on this item by providing additional information not in the ms, or briefly explain why the item is not applicable/relevant for your study

"Informed consent was obtained over the phone and confirmed in person during clinical evaluations. Participants received general information about study activities, including educational content delivery, survey completion at three time points, and their right to withdraw at any time. Specific study aims were withheld to avoid bias, per CIOMS Guideline 10[68]. "

X26-iii) Safety and security procedures

Safety and security procedures, incl. privacy considerations, and any steps taken to reduce the likelihood or detection of harm (e.g., education and training, availability of a hotline)

	1	2	3	4	5	
subitem not at all important	○	○	○	○	○	essential

Does your paper address subitem X26-iii?

Copy and paste relevant sections from the manuscript (include quotes in quotation marks "like this" to indicate direct quotes from your manuscript), or elaborate on this item by providing additional information not in the ms, or briefly explain why the item is not applicable/relevant for your study

Tu respuesta

RESULTS

13a) For each group, the numbers of participants who were randomly assigned, received intended treatment, and were analysed for the primary outcome
NPT: The number of care providers or centers performing the intervention in each group and the number of patients treated by each care provider in each center

Does your paper address CONSORT subitem 13a? *

Copy and paste relevant sections from the manuscript (include quotes in quotation marks "like this" to indicate direct quotes from your manuscript), or elaborate on this item by providing additional information not in the ms, or briefly explain why the item is not applicable/relevant for your study

"Between March 3 and July 20, 2023, 120 participants were randomly assigned to either the telehealth education group (n= 61) or the comparator group (n = 59) (Figure 1). The last patient´s follow-up was completed on September 15, 2023. All patients provided informed consent after enrolling in the trial. The analyses were conducted using data from participants who completed the intervention (n=103), of whom 52 were in the telehealth education group and 51 in the comparator group."

13b) For each group, losses and exclusions after randomisation, together with reasons

Does your paper address CONSORT subitem 13b? (NOTE: Preferably, this is shown in a CONSORT flow diagram) *

Copy and paste relevant sections from the manuscript (include quotes in quotation marks "like this" to indicate direct quotes from your manuscript), or elaborate on this item by providing additional information not in the ms, or briefly explain why the item is not applicable/relevant for your study

It is addressed in the CONSORT flow diagram

13b-i) Attrition diagram

Strongly recommended: An attrition diagram (e.g., proportion of participants still logging in or using the intervention/comparator in each group plotted over time, similar to a survival curve) or other figures or tables demonstrating usage/dose/engagement.

	1	2	3	4	5	
subitem not at all important	○	○	○	○	○	essential

Does your paper address subitem 13b-i?

Copy and paste relevant sections from the manuscript or cite the figure number if applicable (include quotes in quotation marks "like this" to indicate direct quotes from your manuscript), or elaborate on this item by providing additional information not in the ms, or briefly explain why the item is not applicable/relevant for your study

Tu respuesta

14a) Dates defining the periods of recruitment and follow-up

Does your paper address CONSORT subitem 14a? *

Copy and paste relevant sections from the manuscript (include quotes in quotation marks "like this" to indicate direct quotes from your manuscript), or elaborate on this item by providing additional information not in the ms, or briefly explain why the item is not applicable/relevant for your study

"Between March 3 and July 20, 2023, 120 participants were randomly assigned to either the telehealth education group (n= 61) or the comparator group (n = 59) (Figure 1). The last patient´s follow-up was completed on September 15, 2023"

14a-i) Indicate if critical “secular events” fell into the study period

Indicate if critical “secular events” fell into the study period, e.g., significant changes in Internet resources available or “changes in computer hardware or Internet delivery resources”

	1	2	3	4	5	
subitem not at all important	O	O	O	O	O	essential

Does your paper address subitem 14a-i?

Copy and paste relevant sections from the manuscript (include quotes in quotation marks "like this" to indicate direct quotes from your manuscript), or elaborate on this item by providing additional information not in the ms, or briefly explain why the item is not applicable/relevant for your study

Tu respuesta

14b) Why the trial ended or was stopped (early)

Does your paper address CONSORT subitem 14b? *

Copy and paste relevant sections from the manuscript (include quotes in quotation marks "like this" to indicate direct quotes from your manuscript), or elaborate on this item by providing additional information not in the ms, or briefly explain why the item is not applicable/relevant for your study

Not applicable, as the trial period has been completed.

15) A table showing baseline demographic and clinical characteristics for each group

NPT: When applicable, a description of care providers (case volume, qualification, expertise, etc.) and centers (volume) in each group

Does your paper address CONSORT subitem 15? *

Copy and paste relevant sections from the manuscript (include quotes in quotation marks "like this" to indicate direct quotes from your manuscript), or elaborate on this item by providing additional information not in the ms, or briefly explain why the item is not applicable/relevant for your study

Table 1 shows baseline demographic and clinical characteristics for each group

15-i) Report demographics associated with digital divide issues

In ehealth trials it is particularly important to report demographics associated with digital divide issues, such as age, education, gender, social-economic status, computer/Internet/ehealth literacy of the participants, if known.

	1	2	3	4	5	
subitem not at all important	○	○	○	○	◉	essential

Borrar selección

Does your paper address subitem 15-i? *

Copy and paste relevant sections from the manuscript (include quotes in quotation marks "like this" to indicate direct quotes from your manuscript), or elaborate on this item by providing additional information not in the ms, or briefly explain why the item is not applicable/relevant for your study

"Among the assessed for eligibility population, 25% did not use WhatsApp (105/420) (Figure 1), with this trend being more pronounced in rural areas. No significant differences were observed between the intervention groups (P=.86). However, significant differences in smartphone usage were observed based on participants' place of residence (P<.001). Rural residents demonstrated lower engagement with online searches (P=.04), other social media app (P=.042), and email (P<.001), while 45% of urban participants (24/53) engaged in advanced smartphone use (WhatsApp, online search, other social media, and email) (Table 1). Notably, older adults with limited smartphone skills- specifically those using only WhatsApp- were more likely to have eight years of education or less and predominantly lived in rural areas (P=.008). "

16) For each group, number of participants (denominator) included in each analysis and whether the analysis was by original assigned groups

16-i) Report multiple “denominators” and provide definitions

Report multiple “denominators” and provide definitions: Report N’s (and effect sizes) “across a range of study participation [and use] thresholds” [1], e.g., N exposed, N consented, N used more than x times, N used more than y weeks, N participants “used” the intervention/comparator at specific pre-defined time points of interest (in absolute and relative numbers per group). Always clearly define “use” of the intervention.

	1	2	3	4	5	
subitem not at all important	○	○	○	○	◉	essential

Borrar selección

Does your paper address subitem 16-i? *

Copy and paste relevant sections from the manuscript (include quotes in quotation marks "like this" to indicate direct quotes from your manuscript), or elaborate on this item by providing additional information not in the ms, or briefly explain why the item is not applicable/relevant for your study

"The analysis showed that, after six weeks, participants in the Telehealth education reinforcement group (52/103) were significantly more likely than those in the Comparator group (51/103) to demonstrate improved knowledge about dental caries (r= 0.21, 95% CI 0.11-0.3; P=.036) and more positive attitudes toward oral health (r=0.28, 95% CI 0.18- 0.38; P=.004), with small and moderate effect sizes, respectively"

16-ii) Primary analysis should be intent-to-treat

Primary analysis should be intent-to-treat, secondary analyses could include comparing only “users”, with the appropriate caveats that this is no longer a randomized sample (see 18-i).

	1	2	3	4	5	
subitem not at all important	○	○	○	○	○	essential

Does your paper address subitem 16-ii?

Copy and paste relevant sections from the manuscript (include quotes in quotation marks "like this" to indicate direct quotes from your manuscript), or elaborate on this item by providing additional information not in the ms, or briefly explain why the item is not applicable/relevant for your study

Tu respuesta

17a) For each primary and secondary outcome, results for each group, and the estimated effect size and its precision (such as 95% confidence interval)

Does your paper address CONSORT subitem 17a? *

Copy and paste relevant sections from the manuscript (include quotes in quotation marks "like this" to indicate direct quotes from your manuscript), or elaborate on this item by providing additional information not in the ms, or briefly explain why the item is not applicable/relevant for your study

"The comparator group (face-to-face education) showed significant improvements across various oral health outcomes. Notably, the intervention demonstrates large effects in oral health knowledge (Cohen d=0.93, 95% CI 0.52-1.34; P<.001), oral cancer knowledge (r=0.63, 95% CI 0.36-0.90], P<.001), oral health self-efficacy (r=0.62, 95% CI 0.34-0.89; P<.001), and brushing behavior (r=0.51, 95% CI 0.24-0.79; P<.001). Additionally, it had a moderate effect on periodontal disease knowledge (r=0.44, 95% CI 0.17- 0.71; P=.002). However, attitudes toward oral health (P=.760) and dental caries knowledge (P=.124) did not significantly improve.

17a-i) Presentation of process outcomes such as metrics of use and intensity of use

In addition to primary/secondary (clinical) outcomes, the presentation of process outcomes such as metrics of use and intensity of use (dose, exposure) and their operational definitions is critical. This does not only refer to metrics of attrition (13-b) (often a binary variable), but also to more continuous exposure metrics such as “average session length”. These must be accompanied by a technical description how a metric like a “session” is defined (e.g., timeout after idle time) [1] (report under item 6a).

	1	2	3	4	5	
subitem not at all important	○	○	○	◉	○	essential

Borrar selección

Does your paper address subitem 17a-i?

Copy and paste relevant sections from the manuscript (include quotes in quotation marks "like this" to indicate direct quotes from your manuscript), or elaborate on this item by providing additional information not in the ms, or briefly explain why the item is not applicable/relevant for your study

"In terms of overall engagement, there was a 14.2% (17/120) dropout rate during the study´s execution. Notably, at the 6-week mark, there was no statistically significant difference in dropout rates between the telehealth education group (9/61, 15%) and the comparator group (8/59, 13%) (P=.851). Of the 120 participants, 5 (approximately 4%) were excluded from the final analysis due to difficulties engaging with the telehealth intervention. Specifically, these participants reported challenges in accessing and viewing the educational content, despite initially indicating familiarity with using WhatsApp and watching videos. Notably, 4 of these 5 participants who were unable to view the videos were from rural areas. "

17b) For binary outcomes, presentation of both absolute and relative effect sizes is recommended

Does your paper address CONSORT subitem 17b? *

Copy and paste relevant sections from the manuscript (include quotes in quotation marks "like this" to indicate direct quotes from your manuscript), or elaborate on this item by providing additional information not in the ms, or briefly explain why the item is not applicable/relevant for your study

"Participants in the telehealth group were 26% more likely to report flossing compared to the comparator group (RR: 1.26, 95% CI [0.93–1.7], $\chi 2=0.09$), corresponding to an absolute increase in flossing prevalence of 14.3% (RD: 0.14, 95% CI [-0.04–0.33]); however, this effect was not statistically significant. Among rural participants, the telehealth group demonstrated a significantly higher flossing prevalence compared to the comparator group ($\chi 2=0.039$), with an 18.8% increase (RD: 0.188, 95% CI [0.013–0.363]) and participants being 87% more likely to report flossing (RR: 1.87, 95% CI [1.01–3.48])."

18) Results of any other analyses performed, including subgroup analyses and adjusted analyses, distinguishing pre-specified from exploratory

Does your paper address CONSORT subitem 18? *

Copy and paste relevant sections from the manuscript (include quotes in quotation marks "like this" to indicate direct quotes from your manuscript), or elaborate on this item by providing additional information not in the ms, or briefly explain why the item is not applicable/relevant for your study

"Cluster Analysis of Demographic and Intervention-Related Variables in the Telehealth Education Group."

18-i) Subgroup analysis of comparing only users

A subgroup analysis of comparing only users is not uncommon in ehealth trials, but if done, it must be stressed that this is a self-selected sample and no longer an unbiased sample from a randomized trial (see 16-iii).

	1	2	3	4	5	
subitem not at all important	○	○	○	○	○	essential

Does your paper address subitem 18-i?

Copy and paste relevant sections from the manuscript (include quotes in quotation marks "like this" to indicate direct quotes from your manuscript), or elaborate on this item by providing additional information not in the ms, or briefly explain why the item is not applicable/relevant for your study

Tu respuesta

19) All important harms or unintended effects in each group

(for specific guidance see CONSORT for harms)

Does your paper address CONSORT subitem 19? *

Copy and paste relevant sections from the manuscript (include quotes in quotation marks "like this" to indicate direct quotes from your manuscript), or elaborate on this item by providing additional information not in the ms, or briefly explain why the item is not applicable/relevant for your study

No significant harms or unintended effects were reported in either group. The telehealth intervention was conducted using encrypted messaging through WhatsApp, ensuring user privacy.

19-i) Include privacy breaches, technical problems

Include privacy breaches, technical problems. This does not only include physical “harm” to participants, but also incidents such as perceived or real privacy breaches [1], technical problems, and other unexpected/unintended incidents. “Unintended effects” also includes unintended positive effects [2].

	1	2	3	4	5	
subitem not at all important	○	○	○	○	○	essential

Does your paper address subitem 19-i?

Copy and paste relevant sections from the manuscript (include quotes in quotation marks "like this" to indicate direct quotes from your manuscript), or elaborate on this item by providing additional information not in the ms, or briefly explain why the item is not applicable/relevant for your study

No significant harms or unintended effects were reported in either group. The telehealth intervention was conducted using encrypted messaging through WhatsApp, ensuring user privacy.

19-ii) Include qualitative feedback from participants or observations from staff/researchers

Include qualitative feedback from participants or observations from staff/researchers, if available, on strengths and shortcomings of the application, especially if they point to unintended/unexpected effects or uses. This includes (if available) reasons for why people did or did not use the application as intended by the developers.

	1	2	3	4	5	
subitem not at all important	○	○	○	○	○	essential

Does your paper address subitem 19-ii?

Copy and paste relevant sections from the manuscript (include quotes in quotation marks "like this" to indicate direct quotes from your manuscript), or elaborate on this item by providing additional information not in the ms, or briefly explain why the item is not applicable/relevant for your study

Tu respuesta

DISCUSSION

22) Interpretation consistent with results, balancing benefits and harms, and considering other relevant evidence

NPT: In addition, take into account the choice of the comparator, lack of or partial blinding, and unequal expertise of care providers or centers in each group

22-i) Restate study questions and summarize the answers suggested by the data, starting with primary outcomes and process outcomes (use)

Restate study questions and summarize the answers suggested by the data, starting with primary outcomes and process outcomes (use).

	1	2	3	4	5	
subitem not at all important	○	○	○	○	○	essential

Does your paper address subitem 22-i? *

Copy and paste relevant sections from the manuscript (include quotes in quotation marks "like this" to indicate direct quotes from your manuscript), or elaborate on this item by providing additional information not in the ms, or briefly explain why the item is not applicable/relevant for your study

"The primary question was whether telehealth reinforcement could improve oral health knowledge attitudes, and oral health self-efficacy among older adults. The results suggest the effectiveness of both interventions in improving several oral health guides. However, the telehealth education group achieved significantly greater improvements in specific domains, particularly, dental caries knowledge and attitudes toward oral health."

22-ii) Highlight unanswered new questions, suggest future research

Highlight unanswered new questions, suggest future research.

	1	2	3	4	5	
subitem not at all important	○	○	○	○	●	essential

Borrar selección

Does your paper address subitem 22-ii?

Copy and paste relevant sections from the manuscript (include quotes in quotation marks "like this" to indicate direct quotes from your manuscript), or elaborate on this item by providing additional information not in the ms, or briefly explain why the item is not applicable/relevant for your study

Future research is suggested

20) Trial limitations, addressing sources of potential bias, imprecision, and, if relevant, multiplicity of analyses

20-i) Typical limitations in ehealth trials

Typical limitations in ehealth trials: Participants in ehealth trials are rarely blinded. Ehealth trials often look at a multiplicity of outcomes, increasing risk for a Type I error. Discuss biases due to non-use of the intervention/usability issues, biases through informed consent procedures, unexpected events.

	1	2	3	4	5	
subitem not at all important	○	○	○	○	◉	essential

Borrar selección

Does your paper address subitem 20-i? *

Copy and paste relevant sections from the manuscript (include quotes in quotation marks "like this" to indicate direct quotes from your manuscript), or elaborate on this item by providing additional information not in the ms, or briefly explain why the item is not applicable/relevant for your study

"Lastly, the study did not track how frequently participants engaged with the educational videos, which would have provided insights into the potential impact of content engagement on health outcomes. "

21) Generalisability (external validity, applicability) of the trial findings

NPT: External validity of the trial findings according to the intervention, comparators, patients, and care providers or centers involved in the trial

21-i) Generalizability to other populations

Generalizability to other populations: In particular, discuss generalizability to a general Internet population, outside of a RCT setting, and general patient population, including applicability of the study results for other organizations

	1	2	3	4	5	
subitem not at all important	○	○	○	◉	○	essential

Borrar selección

Does your paper address subitem 21-i?

Copy and paste relevant sections from the manuscript (include quotes in quotation marks "like this" to indicate direct quotes from your manuscript), or elaborate on this item by providing additional information not in the ms, or briefly explain why the item is not applicable/relevant for your study

"Moreover, despite efforts to ensure diversity, selection biases related to participants' access to technology or their motivation to engage in the teledentistry program may have influenced the outcomes"

21-ii) Discuss if there were elements in the RCT that would be different in a routine application setting

Discuss if there were elements in the RCT that would be different in a routine application setting (e.g., prompts/reminders, more human involvement, training sessions or other co-interventions) and what impact the omission of these elements could have on use, adoption, or outcomes if the intervention is applied outside of a RCT setting.

	1	2	3	4	5	
subitem not at all important	○	○	○	○	○	essential

Does your paper address subitem 21-ii?

Copy and paste relevant sections from the manuscript (include quotes in quotation marks "like this" to indicate direct quotes from your manuscript), or elaborate on this item by providing additional information not in the ms, or briefly explain why the item is not applicable/relevant for your study

"While the Telehealth reinforcement showed statistically significant but modest effects, these results are particularly meaningful for vulnerable populations, such as older adults in rural areas, where access to face-to-face services is limited. In these groups, even small improvements could lead to cumulative benefits, such as the prevention of more costly oral and general health complications. However, its overall effectiveness should be critically assessed, as its impact appears marginal in individuals with better initial oral health, which may limit its universal applicability. Public health policymakers should consider expanding this intervention model to diverse contexts. Recommending its use will depend on a contextualized cost-effectiveness analysis, prioritizing its implementation in areas of higher need."

OTHER INFORMATION

23) Registration number and name of trial registry

Does your paper address CONSORT subitem 23? *

Copy and paste relevant sections from the manuscript (include quotes in quotation marks "like this" to indicate direct quotes from your manuscript), or elaborate on this item by providing additional information not in the ms, or briefly explain why the item is not applicable/relevant for your study

ClinicalTrials.gov NCT05917548

24) Where the full trial protocol can be accessed, if available

Does your paper address CONSORT subitem 24? *

Cite a Multimedia Appendix, other reference, or copy and paste relevant sections from the manuscript (include quotes in quotation marks "like this" to indicate direct quotes from your manuscript), or elaborate on this item by providing additional information not in the ms, or briefly explain why the item is not applicable/relevant for your study

No full trial protocol available

25) Sources of funding and other support (such as supply of drugs), role of funders

Does your paper address CONSORT subitem 25? *

Copy and paste relevant sections from the manuscript (include quotes in quotation marks "like this" to indicate direct quotes from your manuscript), or elaborate on this item by providing additional information not in the ms, or briefly explain why the item is not applicable/relevant for your study

"funded by the Presidential Grant Project Clinical Center for Innovation in Oral Health of the Elderly in La Araucanía region (Exempt Resolution No. 472).

X27) Conflicts of Interest (not a CONSORT item)

X27-i) State the relation of the study team towards the system being evaluated

In addition to the usual declaration of interests (financial or otherwise), also state the relation of the study team towards the system being evaluated, i.e., state if the authors/evaluators are distinct from or identical with the developers/sponsors of the intervention.

	1	2	3	4	5	
subitem not at all important	○	○	○	○	○	essential

Does your paper address subitem X27-i?

Copy and paste relevant sections from the manuscript (include quotes in quotation marks "like this" to indicate direct quotes from your manuscript), or elaborate on this item by providing additional information not in the ms, or briefly explain why the item is not applicable/relevant for your study

Tu respuesta

About the CONSORT EHEALTH checklist

As a result of using this checklist, did you make changes in your manuscript? *

- ○ yes, major changes
- ◉ yes, minor changes
- ○ no

What were the most important changes you made as a result of using this checklist?

Tu respuesta

How much time did you spend on going through the checklist INCLUDING making changes in your manuscript *

Almost 3 hours to complete the checklist

As a result of using this checklist, do you think your manuscript has improved? *

- (x) yes
- ( ) no
- ( ) Otro:

Would you like to become involved in the CONSORT EHEALTH group?

This would involve for example becoming involved in participating in a workshop and writing an "Explanation and Elaboration" document

- ( ) yes
- (x) no
- ( ) Otro:

Borrar selección

Any other comments or questions on CONSORT EHEALTH

Tu respuesta

STOP - Save this form as PDF before you click submit

To generate a record that you filled in this form, we recommend to generate a PDF of this page (on a Mac, simply select "print" and then select "print as PDF") before you submit it.

When you submit your (revised) paper to JMIR, please upload the PDF as supplementary file.

Don't worry if some text in the textboxes is cut off, as we still have the complete information in our database. Thank you!

Final step: Click submit !

Click submit so we have your answers in our database!

Enviar Borrar formulario

Nunca envíes contraseñas a través de Formularios de Google.

Este formulario se creó fuera de tu dominio. - Términos del Servicio - Política de Privacidad

Does this form look suspicious? Informe

Google Formularios
