# Supplementary material for: WhatsApp-Supported Teledentistry to Reinforce Oral Health Promotion Among Older Adults Residing in Rural and Urban Areas: Randomized Controlled Trial
Source: JMIR Mhealth Uhealth. 2026 May 15;14:e71251. doi: 10.2196/71251 (PMC13221623; doi:10.2196/71251)
Supplement: Multimedia Appendix 2 [file mhealth_v14i1e71251_app2.pdf]

Multimedia Appendix: Baseline characteristics by group

Characteristic	Comparator (n=51)	Telehealth education (n=52)	P value
**Demographics**			
Age (mean (SD))	69.1 (6.2)	68.9 (7.1)	.55[a]
Sex (%female(n))	56 (29)	69 (36)	.19
Education level:			
Low (% ≤8 years(n))	29 (15)	32 (17)	
Secondary (%9-12 years (n))	47 (24)	48 (21)	.56
Technical or university (% ≥ 13 years (n))	23 (12)	27 (14)	
RSH (% RSH≤40 (n))	59 (30)	46 (24)	.86
Smartphone usage level:			
Basic Use (%(n))	18 (9)	21 (11)	.85
Advanced Use (%(n))	31 (16)	27 (14)	
**Medico-geriatric assessment**			
Multimorbidity (%(n))	47 (24)	60 (31)	.20
Depression (%(n))	18 (9)	6 (3)	.07
Frailty Phenotype			
Robust (%(n))	16 (8)	10 (5)	
Prefrail (%(n))	63 (32)	73 (38)	.52
Frail (%(n))	21 (11)	17 (9)	
**Dental assessment**			
Last dental visit:			
Never or more than 10 years (%(n))	16 (10)	14 (7)	
2-10 years (%(n))	32 (23)	39 (20)	.57
≤ 1year (%(n))	35 (18)	47 (24)	
Bad oral health perception (% (n))	24 (12)	47 (24)	.11
Number of remaining teeth (mean (SD))	17.3(7.4)	17.9(6.9)	.65
Toothbrushing frequency:			
0-1 times per day (%(n))	8 (4)	10 (5)	
2 times per day (%(n))	29 (15)	44 (23)	.34
≥3 times per day (%(n))	63 (32)	46 (24)	

[a] Kruskal-Wallis test

This is a Multimedia Appendix to a full manuscript published in the J Med Internet Res. For full copyright and citation information see http://dx.doi.org/10.2196/71251
