# Supplementary material for: WhatsApp-Supported Teledentistry to Reinforce Oral Health Promotion Among Older Adults Residing in Rural and Urban Areas: Randomized Controlled Trial
Source: JMIR Mhealth Uhealth. 2026 May 15;14:e71251. doi: 10.2196/71251 (PMC13221623; doi:10.2196/71251)
Supplement: Multimedia Appendix 3 [file mhealth_v14i1e71251_app3.pdf]

Multimedia Appendix: Baseline characteristics by group and place of residence

Characteristic	Telehealth education (n=52)		Comparator (n=51)		P value
	Rural (n=28)	Urban (n=24)	Rural (n=22)	Urban (n=29)	
**Demographics**					
Age (mean (SD))	68.6(7.5)	69.5(6.8)	69.4(5.9)	69.0(6.7)	.92[a]
Sex (%female(n))	68 (20)	73 (22)	45 (14)	56 (17)	.54
Education level:					
Low (% ≤8 years(n))	34 (10)	30 (9)	32 (10)	26 (8)	
Secondary (%9-12 years (n))	48 (14)	33 (10)	54 (17)	43 (13)	.65
Technical or university (% ≥ 13 years (n))	17 (5)	36 (11)	12% (4)	30 (9)	
RSH (% RSH≤40 (n))	58 (17)	46 (14)	64 (20)	53 (16)	.29
Smartphone usage level:					
Basic Use (%(n))	35 (10)	4 (1)	32 (7)	7 (2)	.002
Advanced Use (%(n))	14 (4)	42 (10)	9 (2)	48 (14)	
**Medico-geriatric assessment**					
Multimorbidity (%(n))	48 (14)	73 (22)	48 (15)	40 (12)	.01
Depression (%(n))	10 (3)	0 (0)	9 (3)	20 (6)	.10
Frailty Phenotype					
Robust (%(n))	10 (3)	13 (4)	9 (3)	16 (5)	
Prefrail (%(n))	75 (22)	60 (18)	64 (20)	70 (21)	.23
Frail (%(n))	13 (4)	26 (8)	19 (6)	13 (4)	
**Dental assessment**					
Last dental visit:					
Never or more than 10 years (%(n))	20 (6)	3 (1)	16 (5)	23 (7)	
2-10 years (%(n))	44 (13)	43 (13)	32 (10)	53 (16)	.18
≤ 1year (%(n))	34 (10)	50 (15)	45 (14)	23 (7)	
Bad oral health perception (% (n))	34 (10)	26 (8)	48 (15)	30 (9)	.23
Number of remaining teeth	15.5(7.2)	20.4(6.1)	16.9(7.2)	18.0(8.0)	.09
Toothbrushing frequency:					
0-1 times per day (%(n))	10 (3)	10 (3)	6 (2)	10 (3)	
2 times per day (%(n))	41 (12)	46 (14)	38 (12)	26 (8)	.63
≥3 times per day (%(n))	48 (14)	43 (13)	48 (15)	63 (19)	

[a] Kruskal-Wallis test

This is a Multimedia Appendix to a full manuscript published in the J Med Internet Res.
For full copyright and citation information see http://dx.doi.org/10.2196/71251
